# HA N193D substitution in the HPAI H5N1 virus alters receptor binding affinity and enhances virulence in mammalian hosts

**DOI:** 10.1080/22221751.2024.2302854

**Published:** 2024-01-08

**Authors:** Seung-Gyu Jang, Young-Il Kim, Mark Anthony B. Casel, Jeong Ho Choi, Ju Ryeon Gil, Rare Rollon, Eun-Ha Kim, Se-Mi Kim, Ho Young Ji, Dong Bin Park, Jungwon Hwang, Jae-Woo Ahn, Myung Hee Kim, Min-Suk Song, Young Ki Choi

**Affiliations:** a College of Medicine and Medical Research Institute, Chungbuk National University, Cheongju, Republic of Korea; b Center for Study of Emerging and Re-emerging Viruses, Korea Virus Research Institute, Institute for Basic Science (IBS), Daejeon, Republic of Korea; c Zoonotic Infectious Diseases Research Center, Chungbuk National University, Cheongju, Republic of Korea; d Microbiome Convergence Research Center, Korea Research Institute of Bioscience and Biotechnology (KRIBB), Daejeon, Republic of Korea

**Keywords:** HPAI H5N8 virus, Clade 2.3.4.4, reassortant, pathogenecity, South Korea, Influenza infections

## Abstract

During the 2021/2022 winter season, we isolated highly pathogenic avian influenza (HPAI) H5N1 viruses harbouring an amino acid substitution from Asparagine(N) to Aspartic acid (D) at residue 193 of the hemagglutinin (HA) receptor binding domain (RBD) from migratory birds in South Korea. Herein, we investigated the characteristics of the N193D HA-RBD substitution in the A/CommonTeal/Korea/W811/2021[CT/W811] virus by using recombinant viruses engineered via reverse genetics (RG). A receptor affinity assay revealed that the N193D HA-RBD substitution in CT/W811 increases α2,6 sialic acid receptor binding affinity. The rCT/W811-HA_193N_ virus caused rapid lethality with high virus titres in chickens compared with the rCT/W811-HA_193D_ virus, while the rCT/W811-HA_193D_ virus exhibited enhanced virulence in mammalian hosts with multiple tissue tropism. Surprisingly, a ferret-to-ferret transmission assay revealed that rCT/W811-HA_193D_ virus replicates well in the respiratory tract, at a rate about 10 times higher than that of rCT/W811-HA_193N_, and all rCT/W811-HA_193D_ direct contact ferrets were seroconverted at 10 days post-contact. Further, competition transmission assay of the two viruses revealed that rCT/W811-HA_193D_ has enhanced growth kinetics compared with the rCT/W811-HA_193N_, eventually becoming the dominant strain in nasal turbinates. Further, rCT/W811-HA_193D_ exhibits high infectivity in primary human bronchial epithelial (HBE) cells, suggesting the potential for human infection. Taken together, the HA-193D containing HPAI H5N1 virus from migratory birds showed enhanced virulence in mammalian hosts, but not in avian hosts, with multi-organ replication and ferret-to-ferret transmission. Thus, this suggests that HA-193D change increases the probability of HPAI H5N1 infection and transmission in humans.

## Introduction

Highly pathogenic avian influenza (HPAI) H5 viruses have been continuously isolated from wild birds and domestic poultry since 1996 [1]. During the initial outbreak in China and Hong Kong in 1997, HPAI H5N1 appeared to be limited to poultry, causing high mortality and resulting in serious economic losses, with only occasional human infections [2–4]. Following its re-emergence in Southeast Asia in 2003, HPAI H5N1 spread to other regions through the migration of infected birds, poultry trade, and human travel. The virus reached Europe, the Middle East, and Africa in 2005, resulting in huge economic damage to the domestic poultry industry [5–7]. Furthermore, the HPAI H5N1 viruses were contemporarily panzootic across three continents, causing human infections with a case fatality rate of ∼53% [8]. Eradication of HPAI virus-infected poultry has been a successful temporary measure to prevent human exposure to these deadly viruses; however, in nature, migratory birds, particularly duck and geese species, are asymptomatically infected with these avian influenza viruses making complete eradication impossible [9,10].

Due to the imprecision of polymerases and the fragmented nature of genomes, HPAI H5N1 viruses have evolved into multiple hemagglutinin (HA) genetic clades and have reassorted with several neuraminidase and internal genes from other Avian Influenza viruses (AIV), resulting in the generation of novel and diverse clades of H5Nx subtypes [11]. For example, in South Korea, the diversity of the clade 2.3.4.4 HPAI H5 viruses resulted in two distinct groups, group A (A/Bdk/Korea/Buan2/2014(H5N8)-like) and group B (A/Brk/Korea/Gochang1/2014(H5N8)-like), wherein group A or 2.3.4.4c emerged as the major strain and group B or 2.3.4.4b as a minor strain [12]. Although both groups caused outbreaks in South Korea in 2014, the 2.3.4.4a H5 viruses spread to East Asia and almost simultaneously to North America and Europe during the 2014–2015 winter season, whereas, 2.3.4.4b caused devastating outbreaks in Asia, Europe, and Africa in 2016–2017 [13–16]. Furthermore, as the 2.3.4.4 undergoes global transmission, almost simultaneously, this virus clade has continuously evolved generating additional groups, known as clade 2.3.4.4c to h. Of these, the 2.3.4.4 group B HPAI H5Nx viruses spread into Europe during the autumnal migration in 2016 and have continuously reassorted with Eurasian low pathogenic avian influenza (LPAI) viruses generating several reassortants including H5N5 and H5N6 HPAI viruses [17,18]. Clade 2.3.4.4b H5N6 and H5N8 HPAI viruses have been continuously detected in Europe, East Asia, and Africa from 2018-2020, and a surge in HPAI H5N8 virus outbreaks occurred in the fall of 2020 in European and Asian countries [19–21].

In the winter of 2021-2022, 2.3.4.4b HPAI H5N1 viruses were isolated from wild bird fecal specimens in South Korea during our routine surveillance. The recent intermittent outbreaks of various HPAI H5Nx viruses in several countries and the emergence of novel HPAI H5Nx subtypes underscore the need for in-depth genetic characterization to investigate the evolutionary dynamics of H5N1. In this study, we genetically characterized the 2021/22 HPAI H5N1 viruses in South Korea and found a distinct mutation resulting in the substitution of Asparagine to Aspartic acid at residue 193 of the receptor binding site (RBD) within the HA glycoprotein. Variations at position 193 of the HA protein exhibit profound implications for influenza viruses, notably, mutations at this position were reported to have an effect on virus adaptability across influenza subtypes, including H5, H3, H7, H9, and H10 [22–27]. The presence of mutations in the HA-193 confers a shift in the receptor binding preference of the virus α2,3 to α2,6 sialic acid or vice versa. However, only limited information concerning the role of Asparagine (N) to Aspartic acid (D) mutation at position HA-193 in pathogenicity, virulence, and transmissibility in mammalian hosts has been reported*.* However, only limited information concerning the role of this mutation in pathogenicity, virulence, and transmissibility in mammalian hosts has been reported. Therefore, we performed detailed *in vitro* and *in vivo* analyses to understand the impact of this mutation. The results revealed that the N193D substitution in the HA RBD altered the binding preference of the virus from avian-like to human-like receptors. More importantly, N193D has a profound effect on growth kinetics and virulence in chicken, mouse, and ferret models, as well as in human respiratory organoids. Our findings highlight the importance of continuous and detailed monitoring as mutations in avian influenzas viruses found in nature pose a great threat to human public health.

## Materials and methods

### Sample collection and virus isolation

Wild bird fecal specimens were collected between October and December of 2021 from wild bird habitats in South Korea. The collected fecal samples were resuspended in antibiotic solution and thoroughly mixed by vortexing, followed by centrifugation (3000 rpm for 15 min at 4 °C). Subsequently, the supernatants were inoculated into specific pathogen-free (SPF) 10-day-old embryonated chicken eggs and incubated for 48 h at 37°C. Virus isolation was confirmed by hemagglutination assay and multiplex RT–PCR as described previously [28]. Allantoic fluids were aliquoted into cryovials (1 mL each) and stored at −80 °C until use.

### Genetic and phylogenetic analyses

Genetic and phylogenetic analyses were conducted as described previously. Briefly, full-length sequences of H5N1 viruses were obtained by Bionics (Seoul, Korea) using an ABI 3730XL DNA analyzer (Applied Biosystems, USA). Sequences were analyzed and compiled with DNA Star 5.0 (DNASTAR, USA); closely related viruses were identified using Basic Local Alignment Search Tool (BLAST). Complete genome sequences were aligned in Clustal W [29], and molecular analysis between Korean isolates and H5Nx viruses was performed. Phylogenetic trees were generated using Bayesian Markov chain Monte Carlo coalescent analysis to investigate the evolutionary history of H5Nx HA-193D worldwide. For the analyzed gene collection, a total of 382 H5Nx HA genes were used for phylogenetic tree analysis by collecting reference sequences required for H5 clades designation and H5Nx HA-193D sequences reported in GISAID and Genbank (Supplementary Table 4 and 5). Various population dynamics models were investigated using the HKY model and 40 million MCMC steps. Maximum-clade-credibility trees were generated using Tree Annotator from the BEAST package, and FigTree v1.4.4 (available at http://tree.bio.ed.ac.uk) was used for visualization of the annotated trees.

### Docking simulation

Docking simulations for the HA mutants were performed using the Autodock Vina software [30]. The ligand structures (Avian, Sialic acid-(α2,3)-Galactose-*N*-acetylglucosamine; Human, Sialic acid-(α2,6)-Galactose-*N*-acetylglucosamine) were prepared by the ACD chemsketch Freeware (ACD, Labs). Hydrogen atoms of receptors and ligand were added in accordance with only polar atoms and converted to pdbqt files by using the Autodocking tools software [31]. Grids for docking was set up with x = −73.1, y = 25.5, and z = 82.9 at centre x = 24.9, y = 25.0, and z = 24.2. An exhaustiveness value of eight was applied for running software.

### Plasmids and rCT/W811-HA_193N/193D_ viruses generation

The eight gene segments of CT/W811(H5N1) were amplified and cloned into the pHW2000 plasmid vector using a plasmid-based reverse-genetics system [32]. To evaluate the HA-RBS N193D substitution, the CT/W811-HA_193N_ virus was generated by site-directed mutagenesis (GeneTailor Site-directed Mutagenesis System; Invitrogen) and compared to CT/W811-HA_193D_. Additionally, we performed full sequencing on both rescued viruses (CT/W811-HA_193N_ and CT/W811-HA_193D_) and viruses pre- and post-utilization in vivo and in vitro studies to ensure they were free of unwanted mutations.

### Receptor binding assays

The receptor-binding preference of the influenza virus isolates was determined using a solid-phase direct virus-binding assay as previously described [33]. Briefly, influenza viruses were bound to fetuin-coated microplates at 4°C overnight. Polyacrylamide (PAA)-biotin-conjugated glycans Neu5Acα2–3Galβ1–4Glc β1 (α2,3’-SL-PAA-biotin) or Neu5Acα2-6Galβ1-4GlcNAc (α2,6’SLN-PAA-biotin) (Glycotech Corporation, USA) were added to influenza-coated plates at varying dilutions and incubated for an additional 4 h. Glycan binding was detected by adding horseradish peroxidase (HRP)-conjugated streptavidin (Invitrogen, USA), and absorbance at 450 nm was measured via a VICTOR3 1420 multilabel-counter plate reader (Perkinelmer, USA). The receptor binding specificities of HPAI H5 viruses were also determined in HA assays using 0.5% re-sialylated chicken red blood cells (cRBCs). For the HA assay, sialic acid residues were enzymatically removed from cRBCs by incubation of the cells with 50 mU Vibrio cholera neuraminidase (VCNA: Roche, San Francisco, CA, USA) at 37 °C for 1 h, the cells were then re-sialyated by incubation with either Neu-5-Ac-α−2,3LacMU(β-D-galactoside α-(2,3)-N-acetylneuraminyltransferase) (Sigma-Aldrich, USA) or Neu-5-Ac-α−2,6-LacMU (α(2,6)-Sialyltransferase from Photobacterium damsela) (Creative enzymes, USA) at 37 °C for 4 h, washed three times in PBS, and re-suspended in PBS containing 1% bovine serum albumin (BSA) [34]. For comparison, positive controls for mammalian (A/California/04/2009 (CA/04, H1N1)) and avian (A/EM/Korea/W795/2020 (EM/W795, H5N8)) influenza viruses for comparing the receptor-binding preferences, and A/mallard duck/Korea/W401/2011 (MDk/W401, H5N1) (a 2.3.2.1c clade avian influenza positive control) were also added as control.

### Cells

Madin-Darby canine kidney (MDCK) cells and chicken fibroblast (DF-1) cells (American Type Culture Collection, USA) were maintained in Eagle's minimum essential medium with Earle's salts (EMEM) (Lonza, Switzerland) containing 10% fetal bovine serum (FBS) (Gibco Life Technologies, USA). Chicken embryo fibroblast (CEF) cells were obtained from 12-day-old SPF chicken embryos. Human lung epithelial Calu-3 and A549 cells were purchased from Korean Cell Line Bank (KCLB) (Seoul, Korea, no. 30055 and 10185), grown and maintained in Dulbecco’s Modified Eagle Medium (DMEM) supplemented with 10% FBS, 100 IU/mL of penicillin, and 100 μg/mL of streptomycin. Primary Human Bronchial Epithelial Cells (HBEpC) were purchased from PromoCell (Heidelberg, Germany) and cultured in an Air–Liquid Interface (ALI) 3D culture system according to the manufacturer's instructions [35] for over 30 days for differentiation into ciliated airway organoids with cilia beating on the outer surface. All cells were incubated at 37°C in 5% CO_2_ until use.

### Virus titrations

Virus titres in virus stocks, oropharyngeal and cloacal swabs, nasal washes, homogenized/clarified tissue samples, and culture supernatants were determined by performing endpoint titrations in SPF 10-day-old embryonated chicken eggs, monolayers of MDCK cells, or both. Eggs or MDCK cells were inoculated with 10-fold serial dilutions of each sample in phosphate-buffered saline solution or fetal bovine serum-free media containing antibiotics. After a 48 h incubation at 37°C, the presence of viruses was detected by a standard HA assay using 0.5% turkey erythrocytes. Mean virus titres are expressed as log_10_EID_50_ or as log_10_TCID_50_ per unit sample (mL or g) tested. The detection limit of EID_50_ is 0.7 log_10_EID_50_/mL, and that of TCID_50_ is 0.8 log_10_TCID_50_/mL [36].

### Animal studies

Chickens, mice, and ferrets used in infection experiments were determined to be influenza-free by hemagglutination inhibition (HI) assay against currently circulating human and/or avian influenza viruses. The experimental animals were arbitrarily allocated to groups and investigators were aware of the allocations. All experiments with H5N1 viruses were conducted in a BSL3 + facility.

### Chicken study

Five-week-old female SPF White Leghorn chickens (CAVac Lab. Co., Ltd., Korea) were used in this study. For intravenous pathogenicity index (IVPI), SPF chickens (n = 10/group) were intravenously inoculated with 0.1 mL of a 1∶10 dilution (using sterile PBS) of allantoic fluid containing >16 HAU of the CT/W811-HA_193N_ and CT/W811-HA_193D_ following the World Organization for Animal Health (WOAH) standards [37]. To evaluate the pathogenic characteristics, chickens (n = 9/group) were infected intranasally with rCT/W811-HA_193N_ or rCT/W811-HA_193D_ with 100 µL of 10^6.0^ EID_50_/mL virus. Tracheal and cloacal swabs were collected from the inoculated birds every day. Three chickens per group were euthanized at 3- and 5-days post-infection (dpi), and organs (brain, lungs, liver, kidney, spleen, and intestines) were harvested for virological examination.

### Mouse study

To determine the 50% mouse lethal dose (MLD_50_) of rCT/W811-HA_193N_ and rCT/W811-HA_193D_, groups of 5-week-old female BALB/c mice (Samtako, Korea) (n = 5/group) were intranasally inoculated with 30 µL of 10^2.0^–10^5.0^ EID_50_/mL of the virus. Baseline body weights were measured prior to infection and survival rates and subsequent body weights were recorded for 14 days after infection. The MLD_50_ was calculated using the method of Reed and Muench [36] and is expressed as EID_50_/mL. For biological and pathological examinations, 18 mice per group were infected intranasally with either rCT/W811-HA_193N_ and rCT/W811-HA_193D_ at 30 µL of 10^6.0^ EID_50_/mL. Six mice were euthanized at 3 and 5 dpi, respectively, a subset of three mice was used to examine virus growth kinetics in mouse lung and extrapulmonary tissues, and the lungs of the remaining three mice were fixed in 4% formalin for RNA scope. Virus titres in various organs were measured by EID_50_ assay.

### Ferret study

To assess virus replication and animal-to-animal transmission following inoculation of CT/W811-HA_193N_ and CT/W811-HA_193D_, influenza A seronegative 16- to 18-week-old ferrets (ID Bio Co., Korea) (n = 11/group) were intranasally inoculated with 10^6.0^ TCID_50_/mL of CT/W811-HA_193N_ or CT/W811-HA_193D_ viruses under anesthesia. For virus transmission studies, each of the inoculated ferrets was co-housed with a naïve direct contact (DC) ferret at 24 h post-infection, and in the same isolator, an indirect contact (IC) ferret was placed adjacent to the inoculated ferret but separated by stainless steel grill dividers to permit aerosol transmission (1:1:1 set-up in triplicate). Bodyweight and temperature of the animals were monitored daily until 10 days. Nasal washes were collected from the directly infected ferrets every other day for 9 days beginning at 1 dpi and daily among contact ferrets from 1-day post-exposure. Ferrets (n = 3/group) were euthanized at 3 and 5 dpi and various organs (brain, lungs, liver, kidney, spleen, and intestines) were collected to assess virus replication. Nasal washes and tissue virus titres were determined by TCID_50_ assay.

### Ethics statement

All animal studies were carried out by protocols approved by the Institutional Animal Care and Use Committee (IACUC) at Chungbuk National University (Approval number CBNUA-1638-21-02). Animal experiments were carried out in an enhanced biosafety level 3 facility at Chungbuk National University as permitted by the KCDC (Permit No.: KCDC-14-3-07).

### Immunohistochemistry (IHC)

IHC staining with Influenza NP in ferret lungs was performed on a Leica Bond RX automated staining instrument using the Bond Polymer Refine Detection System IHC protocol F (Leica Biosystems, UK, DS9800) with the following modifications: 20-min heat-induced epitope retrieval using ER2, 20-min protein block, and 30-min primary antibody incubation at 1/100 dilution. Following staining, slides were dehydrated through graded alcohols, coverslips added, and mounted. Stained slides were digitally scanned using the PhenoImager slide scanner (AKOYA Bioscience, USA) and visualized using PhenoImager software.

### RNA scope *in situ* hybridization

Influenza virus RNA (NP) was detected using the NP-speciﬁc probe (Advanced Cell Diagnostics (ACD), USA, 504141) and visualized using RNAscope 2.5 HD Reagent Kit RED (ACD, USA, 322360). According to the manufacturer’s instructions, mouse lung tissues and ALI cultured HBEpC were fixed in 4% neutral buffered formalin, embedded, sectioned, and counterstained with Gill's hematoxylin #1 (Polysciences, 24242-1000). Slides were viewed using Olympus IX 71 (Olympus, Japan) microscope with DP controller software to capture images. For statistical analysis of *in situ* samples, positive foci (most likely resembling single positive cells) were counted manually for each ﬁeld, and standard deviation was calculated using GraphPad Prism 9 software (Version 9.4.1).

### Next-generation sequencing

**Figure 7.**
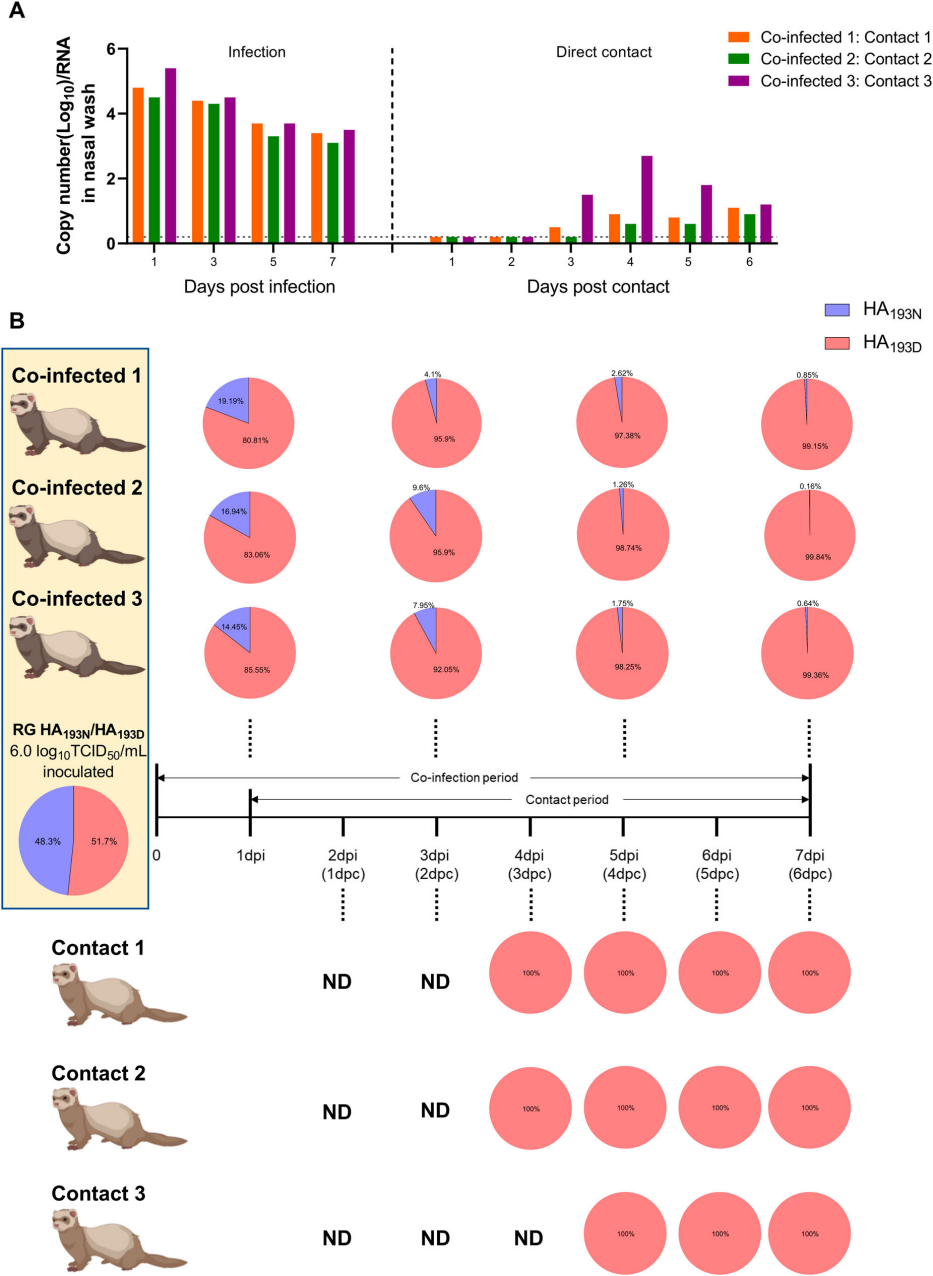
**Competitive replication and transmission of rCT/W811-HA_193N_ and rCT/W811-HA_193D_ viruses.** Infectious viral RNA copy numbers (A) over time in nasal wash samples are shown. Schematic diagram of the experimental setup of three ferret pairs; each co-infected donor ferret having housed together with one naïve direct contact ferret (B). Nasal wash samples were collected every other day from 1 to 7 dpi, and daily among contact ferrets from 1-day post contact (dpc) to 6 dpc. Each pie chart depicts proportions of A or G nucleotides at position 613 (corresponding to N (blue) or D (red) at amino acid position 193, respectively) detected from individual nasal wash samples over time. The limit of virus detection of (A) was 0.2 log_10_ viral RNA copy/0.2 mL (dashed lines).

For virus competition experiments in ferrets, amplicons were sequenced on the Miniseq system from Illumina. Using a RNeasy Kit (Qiagen), total RNA was isolated from ferret nasal washes on the peak shedding day (as described in Figure 7). The first 480–772 bases of the HA gene (containing the receptor binding domain) were amplified by a one-step RT–PCR kit (Qiagen) with gene-specific primers (Forward: 5'-cagaaatgtggtgtggcttat-3’; Reverse: 5'-cggatgatgcaatccatttcg-3’) and subjected to PCR purification (Qiagen). Purified products were subjected to next-generation sequencing, and libraries were created with an Illumina Nextera XT DNA Library Preparation Kit. The libraries were then run on the Miniseq platform (Illumina, USA), using the 2 × 150 kit for a total of ∼300 cycles.

### Immunostaining

HBEpCs were fixed with 4% paraformaldehyde at room temperature (RT) for 24 h. The sections made as Formalin-Fixed Paraffin-Embedded (FFPE) were pre-treated according to the RNAscope multiplex fluorescent reagent kit version 2 (V2) Assay (ACD, USA). Staining was performed on the Leica BOND RX automated stainer with the preloaded modified MOTiF protocol. Briefly, the section was then stained for sialic acid (SA) using lectin staining. Sambucus nigra agglutinin (SNA) primarily detects sialic acid linkages that are α2-6, while Maackia amurensis lectins (MAL)-II identify SA linkages that are α2-3. After influenza H5N1 probe hybridization, the sections were blocked with an avidin/biotin blocking kit (Leica bio, RE7170-K) for 30 min at RT. Samples were incubated with streptavidin HRP (Abcam, ab64269) and signal development was performed using Opal 520, 620, and 690 dye, respectively. The sections were washed with 1X TBS with Spectral DAPI for 5 min. Cover slips were then added with ProLong Gold Antifade Mountant (Invitrogen™, USA) and stored at 4°C until analysis.

### Single nucleotide polymorphism analysis

To investigate the distribution and frequency of various amino acid at HA-193 among H5Nx viruses, HA sequences of H5Nx isolates (23,696) from avian and humans deposited in GISAID and Genbank were collected and analyzed using the SNP analysis tool of the Influenza Virus Database (http://www.fludb.org/).

### Thermal stability test

128 HA units for viruses were incubated at 50°C for the indicated times. Subsequently, infectivity and hemagglutination activity were determined using TCID_50_ assays in MDCK cells and hemagglutination assays using 0.5% turkey RBC, respectively.

### Data analysis

All data analysis and statistical calculations were performed using GraphPad Prism® v9.4.1 (GraphPad Software, Inc.) and are expressed as mean ± SEM. Statistical significance was determined using a two-way ANOVA test with Sidak correction and Kruskal–Wallis test with Dunn’s multiple comparisons test. *p* < 0.05 was considered significant.

## Results

### Genetic characterization of avian influenza A (H5N1) viruses

During the 2021–2022 winter, six HPAI H5N1 viruses were isolated from fecal samples obtained from migratory bird habitats of *Anas Crecca* (*Common Teal*) in South Korea. Meanwhile, full-length genomic sequence analysis revealed that all six H5N1 viruses showed 98.9% to 100% nucleotide homology to one another, and were closely associated with the dominant contemporary HPAI H5N1 viruses isolated from domestic poultry in South Korea [38].

Molecular analysis demonstrated that HA cleavage sites of H5N1 viruses bear polybasic residues (RERRRKR/GLF) denoting a high-pathogenicity phenotype in chickens. The H5N1 isolates maintained the glutamine residue at position 226 (H3 numbering) and a glycine residue at position 228, which is suggestive of preferential binding to sialic acid receptors joined to sugar chains via an α−2,3 linkage, as is typical for avian influenza viruses [39]. Interestingly, two isolates (CT/W811 and CT/W813) of six H5N1 viruses possess an aspartic acid (D) substitution at HA-193 residue in the receptor binding site, which is unique from previously isolated HPAI H5N1 and H5N8 viruses that possess a lysine (K) or asparagine (N) residue (Supplementary Table 1). Compared with previous Korean HPAI A/EM/Korea/W149/06, all 2021–2022 H5N1 viruses have glutamic acid (E) and aspartic acid (D) at position 627 and 701, respectively, which is characteristic of low pathogenicity as per their PB2 genes. However, all isolates contained functional PB1-F2, which have been shown to impact host defense mechanisms and in turn, enhance pathogenicity in mammalian hosts [40]. The H5N1 viruses harbour the 92D in the non-structural 1 (NS1) protein and an ESEV in the C-terminal PDZ-binding motif, wherein both are associated with increased virulence in mice [41,42]. No other mammalian-adaptive molecular determinants were observed in the H5N1 viral genome [43].

**Figure 1.**
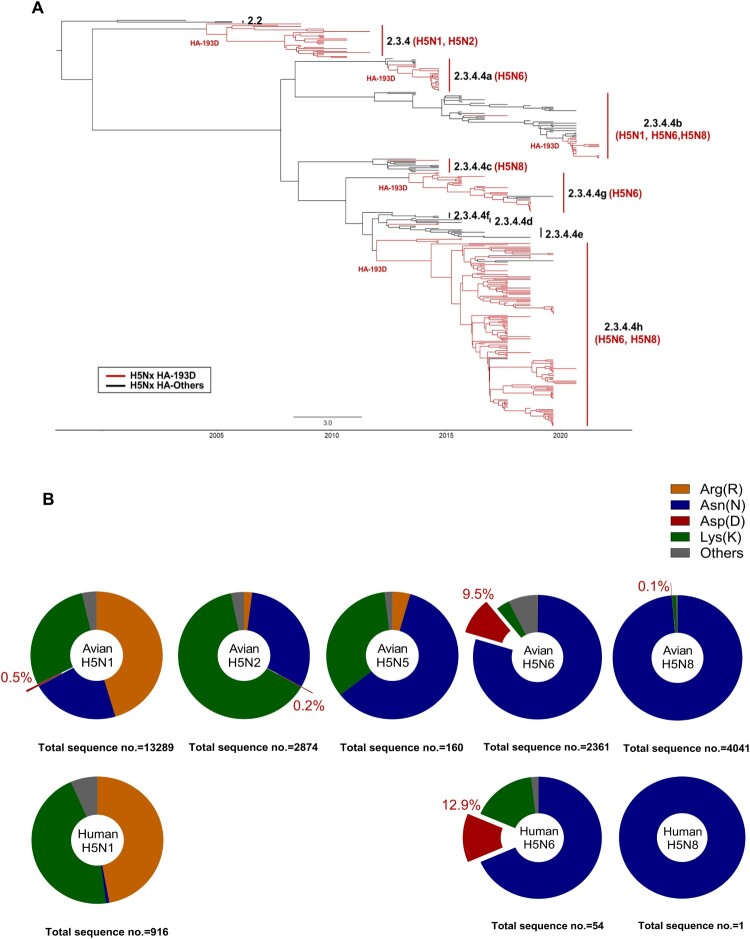
Proportions of amino acid types in influenza H5Nx HA 193 residue and phylogenetic tree analysis of H5Nx influenza with HA_193D_ variants. The phylogenetic tree of H5Nx influenza virus of HA-193D mutation reported from 2007 to 2022, branches and names were represented to clade and H5Nx type (A). Phylogenetic tree analysis of H5Nx influenza with HA-193D variants reported in GISAID and Genbank. A Bayesian Markov Chain Monte Carlo (MCMC) approach using BEAST v.2.7.4 and divergence time estimation using an exponential relaxation clock model were used to reconstruct the phylogenetic tree of 328 H5Nx HA genes. The distribution ratio of varied amino acids at HA-193 in avian or human isolates of the H5Nx influenza virus (B). In the total number of sequences of each H5Nx type, the proportion of sequences with 193D is shown in red and expressed as a percentage. Avian and human-derived isolates of each H5Nx were indicated, and human-derived H5N2 and H5N5 sequences were not reported.

BEAST analysis was performed to determine the emergence of the 193D among the 2.3.4.4 H5Nx viruses, results revealed that the 193D mutation within the RBD was first isolated from a chicken in Henan in 2007 and has been present in various subclades (a-h) of the 2.3.4.4 clade to date (Figure 1A). Further, to investigate the prevalence of HA N193D substitution among the H5Nx viruses, single nucleotide polymorphism (SNP) analysis was performed with a total of 23,696 H5Nx sequences deposited in the GSAID and GenBank database. Although Arginine (R) or Asparagine (N) are the most prevalent residues in H5N1 or H5Nx viruses, 65 (0.5%) of 13,289 H5N1 and 197 (9.5%) of 2,361 H5N6 viruses have Aspartic acid at residue 193, including ∼12.9% of human H5N6 isolates, suggesting that the HA-193D substitution is a natural event (Figure 1B).

### Comparison of receptor-binding preferences according to conformational changes and thermostability caused by mutation of the HA-193 residue

**Figure 2.**
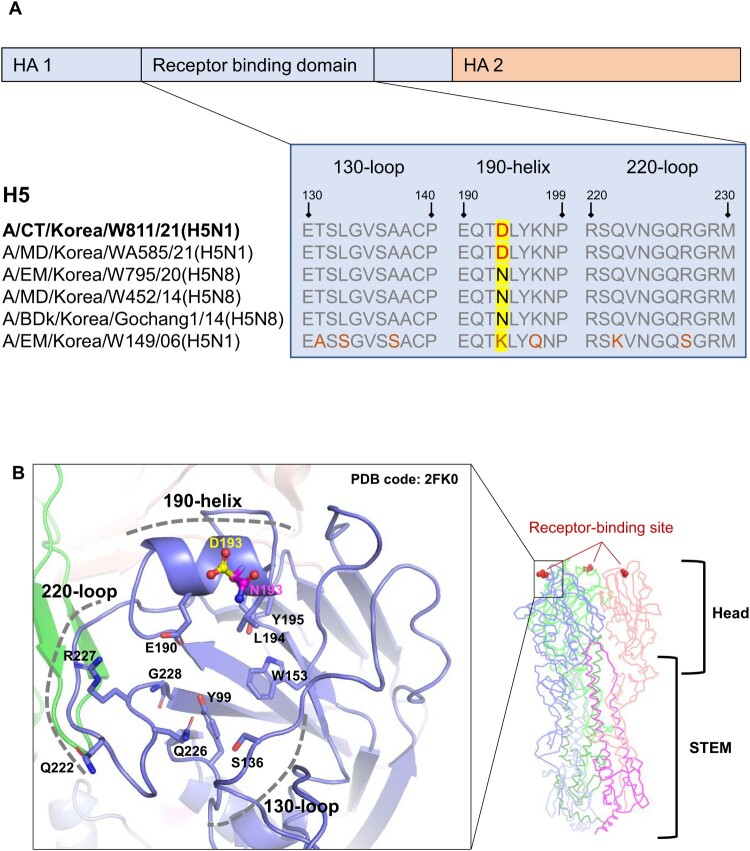
**Receptor binding site of H5 avian influenza hemagglutinin.** Amino acid sequence alignment of the HA 1 130-loop, 190-helix, and 220-loop (receptor binding domain) from pre-H5 isolates and CT/W811 (H5N1) viruses is shown (A). Cartoon structures of the H5 head domain depict the 193-site HA residues altered by amino acid changes, represented in stick form and coloured by element (blue-purple =  carbon, red  =  oxygen, and blue  =  nitrogen). D193 and N193 are highlighted in yellow and magenta, respectively (B). All amino acid residues are presented using H3 numbering. Images were created with Pymol (http://www.pymol.org/).

**Figure 3.**
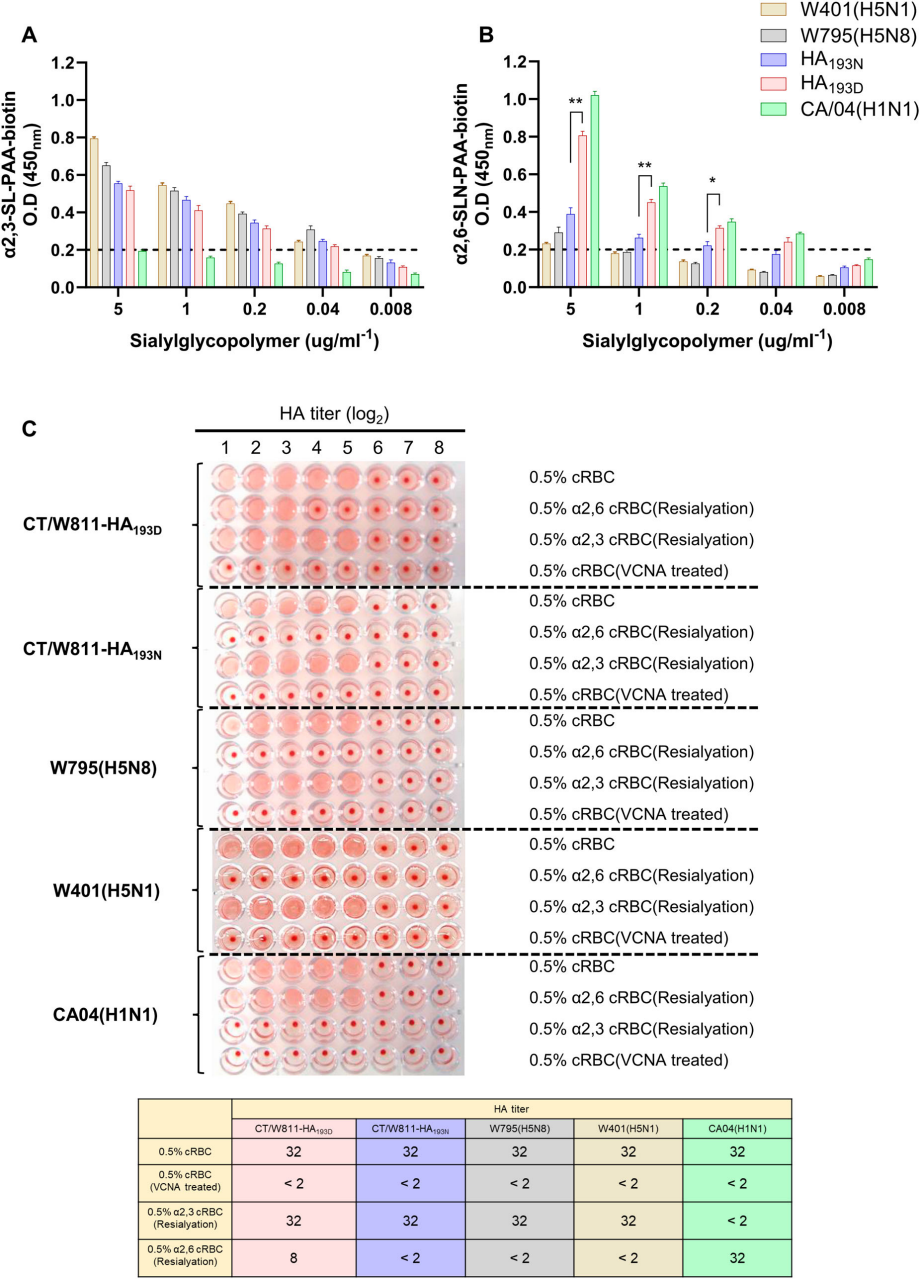
**Receptor-binding preference of rCT/W811-HA_193N_ and rCT/W811-HA_193D._** Binding affinities of inactivated whole viruses to SA α2,3-SL-PAA-biotin (A) and SA α2,6SLN-PAA-biotin (B) glycans. For comparison, the receptor-binding specificity of the A/California/04/2009 (CA/04, H1N1), A/EM/Korea/W795/2020 (EM/W795, H5N8), and A/mallard duck/Korea/W401/2011 (MDk/W401, H5N1) viruses were used as positive controls for mammalian and avian influenza viruses, respectively. HA assays using re-sialylated cRBCs. The results obtained with re-sialylated cRBCs were normalized to the results obtained with untreated cRBCs (C). The results shown are mean ± SEM (mean of three replicates; * indicates *p* < 0.01, and ** indicates *p* < 0.0001). The dashed lines indicate the limit of detection.

Structural analysis revealed that the HA-193 residue is located in the apex head domain of HA1 and forms a receptor-binding pocket (Figure 2). RBD docking simulation with avian or mammalian glycans demonstrated that the N193D substitution within the RBD could induce stronger binding affinity with the mammalian glycan, suggesting that a single amino acid mutation of RBD may alter the receptor-binding tropism and host ranges (Supplementary Figure 1). To validate the results of the RBD docking simulation, we generated a HA-193N substitution virus (rCT/W811-HA_193N_) on the parental CT/W811-HA_193D_ backbone via a plasmid-based reverse genetics (Rg) system. In order to test the effect of structural variation of N193D on receptor binding affinity, we performed solid-phase direct binding assays and measured virus affinity for biotinylated glycans (α 2,3’SA or α 2,6’SLN, which represent the avian and mammalian receptors for these viruses, respectively) [33,34]. The results revealed both the rCT/W811-HA_193N_ and rCT/W811-HA_193D_ viruses demonstrate a high binding affinity for α2,3-linked sialic acids (α2,3-SAs), similar to the control avian A/Environment/Korea/W795/2020(H5N8) [EM/W795] HPAI virus, while the control human A/California/04/2009(H1N1) [CA/04] virus exhibited no binding affinity for α2,3-SAs (Figure 3A). Further, rCT/W811-HA_193N_ and EM/W795(H5N8) showed a lower binding affinity for α2,6-linked sialic acids (α2,6-SAs) compared with the CA/04(H1N1) virus, the positive control for α2,6-SAs binding activity (Figure 3B). It is noteworthy that although the CA/04(H1N1) virus exhibited the strongest binding preference for α2,6-SAs, rCT/W811-HA_193D_ also showed significantly higher binding affinity than the rCT/W811-HA_193N_ for α2,6-SLN-PAA-biotin probes (Figure 3B). We further confirmed the receptor specificity of these viruses by performing a resialylated red blood cell assay (Figure 3C). The rCT/W811-HA_193D_ displayed the expected receptor attachment pattern by binding to both α2,6-SAs and α2,3-SAs, while rCT/W811-HA_193N_ displayed a phenotype similar to typical avian viruses by binding exclusively to α2,3-SAs, further corroborating the results of the solid-phase binding assay. Since heat treatment is recognized to induce a fusogenic state in the HA protein and is considered a substitute assay for HA stability [44], we next evaluated whether identified HA mutations could influence the heat stability of the HA protein. Results showed that the rCT/W811-HA_193N_ and rCT/W811-HA_193D_ retained their infectivity even after 240 mins of incubation at 50°C (Supplementary Figure 2). Taken together, these results demonstrate that the fine balance between affecting HA functions (dual receptor binding affinity for α2,6-SAs and α2,3-SAs and heat stability) may be crucial for the transmissibility of the rCT/W811-HA_193D_ virus which was also observed in human infectious HPAI H5N1 viruses [44].

### Comparison of viral replication in cell lines

**Figure 4.**
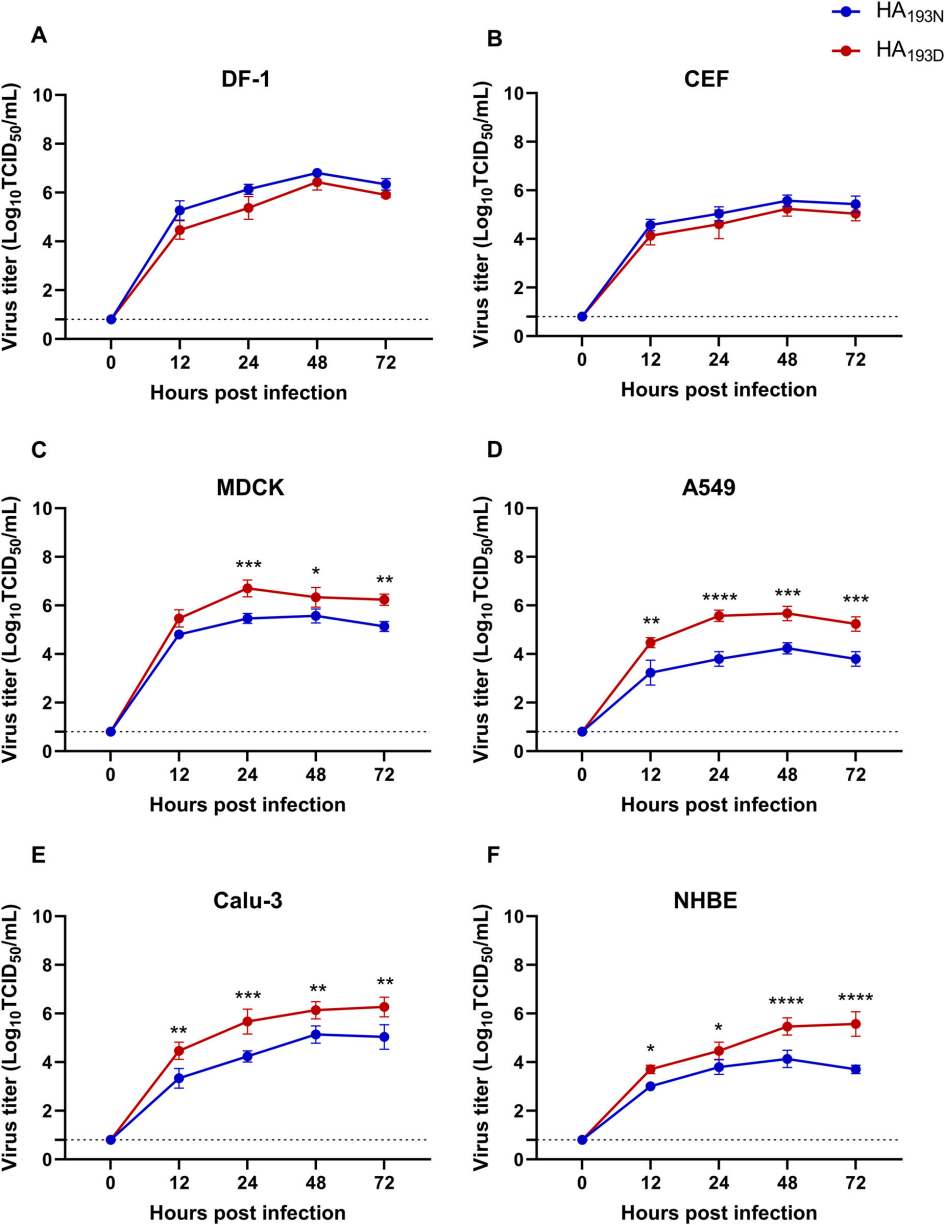
**Growth curves of the rCT/W811-HA_193N_ and rCT/W811-HA_193D_ viruses in avian- and mammalian-origin cells.** Cell monolayers of DF-1 (A), CEF (B), MDCK (C), A549 (D), Calu-3 (E), and NHBE (F) were infected with the rCT/W811-HA_193N_ and rCT/W811-HA_193D_ viruses at an MOI of 0.01 for 72 h. TCID_50_ virus titres were measured in the supernatants at the indicated time points. The data are presented as the mean values of three inoculated wells ± the SEM for each time point and are representative of three independent experiments. * indicates *p* < 0.05, ** indicates *p* < 0.01, *** indicates *p* < 0.001, and **** indicates *p* < 0.0001.

To evaluate if the N193D substitution could affect virus growth kinetics, the viral growth curves of the two viruses (rCT/W811-HA_193N_ and rCT/W811-HA_193D_) were measured in various cell lines. For comparison, avian-origin chicken fibroblast cells (DF-1) and chicken embryo fibroblast (CEF), mammalian-origin Madin-Darby canine kidney cell (MDCK), human lung-origin cells (A549 and Calu-3), and normal human bronchial epithelial (NHBE) cell lines were selected. The rCT/W811-HA_193N_ and rCT/W811-HA_193D_ viruses showed no marked differences at any time point in avian-origin CEF and DF-1 cells (Figure 4A and B). However, the titre of the rCT/W811-HA_193D_ virus was significantly higher than that of rCT/W811-HA_193N_ from 24 hpi up to 72 hpi in all mammalian origin MDCK, A549, Calu-3, and NHBE cells (Figure 4C-F). These results revealed that the substitution of the HA-193 residue from asparagine to aspartic acid could alter the binding affinity, and by extension, the virus growth kinetics, of the virus in mammalian and human-derived cells.

### Comparison of chicken and mouse pathogenicity

**Figure 5.**
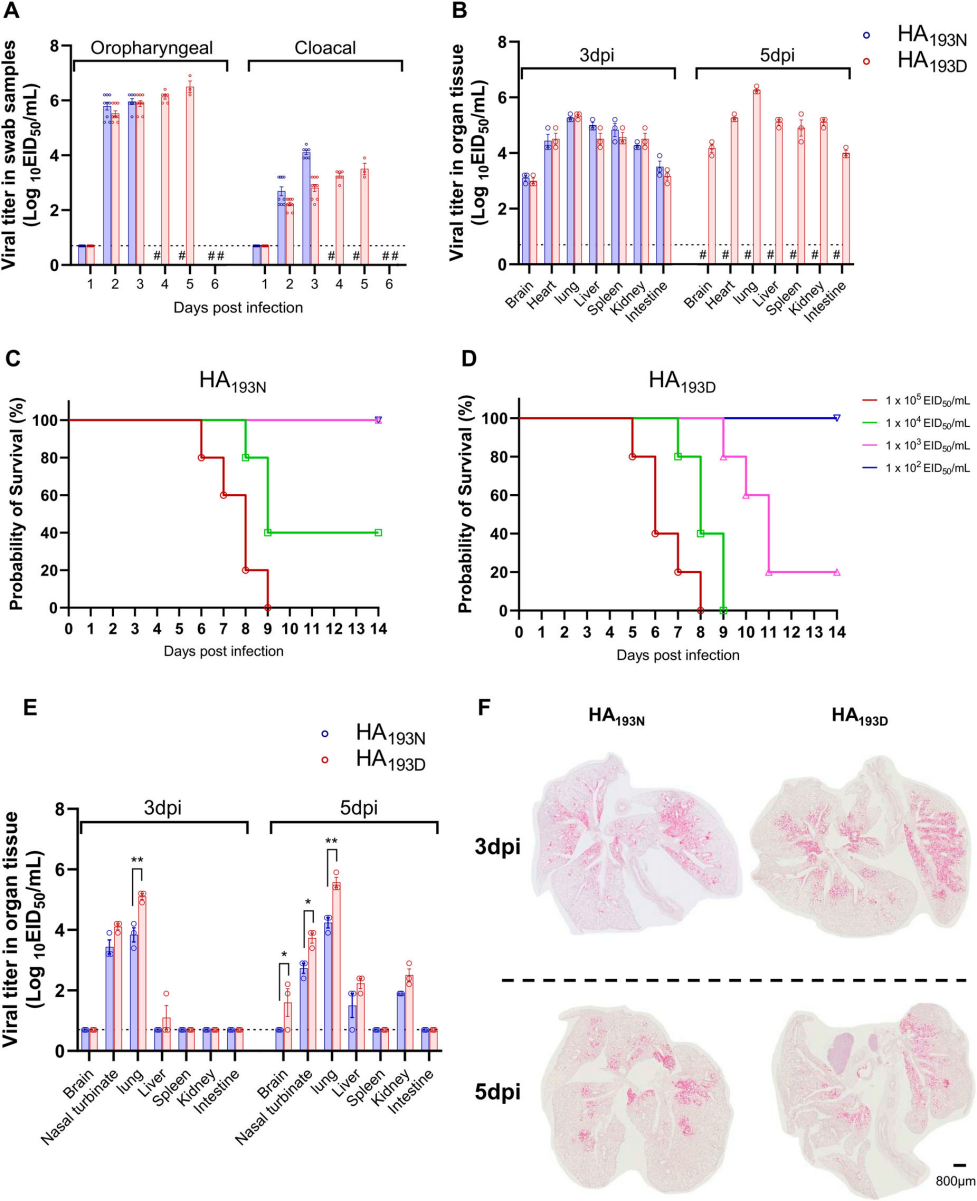
**Replication of rCT/W811-HA_193N_ and rCT/W811-HA_193D_ viruses in chicken and mice.** Comparison of virus replications in experimentally inoculated chickens by the intranasal route with 10^6.0^ EID_50_/0.1mL of each virus. Virus titres of the chicken oropharyngeal and cloacal swabs (A) and in various organ tissues (B). MLD_50_ in mice challenged with serially diluted rCT/W811-HA_193N_ (C) and rCT/W811-HA_193D_ viruses (D). Organ tissue titres were measured and compared in mice inoculated with 10^6.0^ EID_50_/mL of rCT/W811-HA_193N_ and rCT/W811-HA_193D_ viruses, respectively (E). Detection of influenza RNA (NP) in the lungs at 3 and 5days post-infection (dpi) with rCT/W811-HA_193N_ and rCT/W811-HA_193D_ viruses in mice (F). The sharp (#) indicates no samples collected because the chickens in this group died. The limit of virus detection was 0.7 log_10_EID_50_/mL (dashed lines) and viral titres shown are means ± SEM. Magniﬁcation is 1.0x and the scale bar represents 800 µm. * indicates *p* < 0.01, and ** indicates *p* < 0.0001.

To assess the virulence of the rCT/W811-HA_193N_ and rCT/W811-HA_193D_ in animal models, we initially measured the mean death time (MDT) in chickens using IVPI test. Results revealed that both rCT/W811-HA_193N_ and rCT/W811-HA_193D_ viruses showed a 100% mortality with an MDT of 36 h and an IVPI of 2.93 and 2.90, respectively (Supplementary Table 2), suggesting that both viruses can be classified as HPAI according to the WOAH criteria [36]. To further compare the virulence of rCT/W811-HA_193N_ and rCT/W811-HA_193D_ in chickens, groups of 5-week-old SPF chickens (n = 9/group) were inoculated with 10^6.0^ EID_50_/0.1mL with the viruses and viral titres were subsequently measured in oropharyngeal and cloacal swab samples. The rCT/W811-HA_193N_ virus was isolated from 2 until 3 dpi, exhibiting peak virus titres at 2 dpi in both the oropharyngeal and cloacal swabs as 6.9 and 3.9 log_10_EID_50_/mL, respectively (Figure 5A). The remaining chickens succumb to death at 4 dpi. However, the rCT/W811-HA_193D_ virus was continuously recovered from both organs until 5 dpi, exhibiting a gradual increase of virus titres in both the oropharyngeal and cloacal swabs with peak titres of 6.9 and 3.9 log_10_EID_50_/mL at 5 dpi, respectively (Figure 5A), and all reaming chickens died at 6 dpi. In addition, the brain, heart, lungs, liver, kidney, spleen, and intestines were collected from infected chickens (n = 3) at 3 and 5 dpi to determine viral tissue distribution. At 3 dpi, all collected tissues were positive for rCT/W811-HA_193N_ and rCT/W811-HA_193D_ with the highest viral titres observed in the lungs (as high as 5.4 and 5.2 log_10_EID_50_/g, respectively) (Figure 5B). Although we could not compare the virus titres at 5 dpi due to the early death of rCT/W811-HA_193N_ infected chickens, the rCT/W811-HA_193D_ infected chickens showed increased virus titres at 5 dpi in all organs tested compared with 3 dpi. These results demonstrate that the 2021–2022 H5N1 viruses are highly pathogenic to chickens resulting in a systemic infection affecting multiple organs, and that the rCT/W811-HA_193N_ virus causes more early lethality with high virus titres compared with rCT/W811-HA_193D_ virus.

Next, to assess the role of the HA N193D substitution on viral pathogenicity in mice, we initially evaluated the 50% mouse lethal dose (MLD_50_) of each mutant virus. Briefly, groups of mice were infected intranasally with 10^2.0^–10^5.0^ EID_50_/mL of rCT/W811-HA_193N_ and rCT/W811-HA_193D_, followed by a 2-week observation period (Figure 5C and D, Supplementary Figure 3A and B). The results revealed that rCT/W811-HA_193N_ and rCT/W811-HA_193D_ have an MLD_50_ of 10^3.8^ and 10^2.7^ EID_50_/mL, respectively (Figure 5C and D), a 10-fold difference in MLD_50_. Further, to evaluate and compare disease phenotypes of rCT/W811-HA_193N_ and rCT/W811-HA_193D_ viruses, groups of 5-week-old seronegative BALB/c mice (n = 18) were inoculated via the intra nasal route with each of the H5N1 viruses at 10^6.0^ EID_50_/mL. All infected mice showed acute clinical disease manifestation, such as ruffled fur, reduced activity, and inappetence, within 4 days of infection. Both viruses were initially isolated from nasal turbinates and lungs at 3 dpi and subsequently detected in the liver and kidneys at 5 dpi (Figure 5E). Interestingly, the rCT/W811-HA_193D_ infected mice showed higher virus titres in the lungs at 3 dpi and were detected with a broader tissue distribution with high viral titres particularly in the liver, kidney, and brain at 5 dpi (Figure 5E). Meanwhile, all remaining mice (n = 5) succumb to death at 7 dpi. RNA scope staining with NP probes revealed a wider distribution of rCT/W811-HA_193D_ virus in infected mouse lungs compared to that of the rCT/W811-HA_193N_ virus at both 3 and 5 dpi (Figure 5F). These data suggest that the rCT/W811-HA_193D_ virus showed more virulence with high virus titres compared with the rCT/W811-HA_193N_ virus in mice. Taken together, our results demonstrate that despite the delayed mortality exhibited by the rCT/W811-HA_193D_, both rg-viruses displayed high virulence in chickens. Furthermore, rCT/W811-HA_193N_ exhibited significantly enhance pathogenicity and lethality in mouse models, highlighting the key mutation in the RBD that confers the ability of the virus to change host range and enhance virulence.

### Virus replications and transmissibility in ferrets

**Figure 6.**
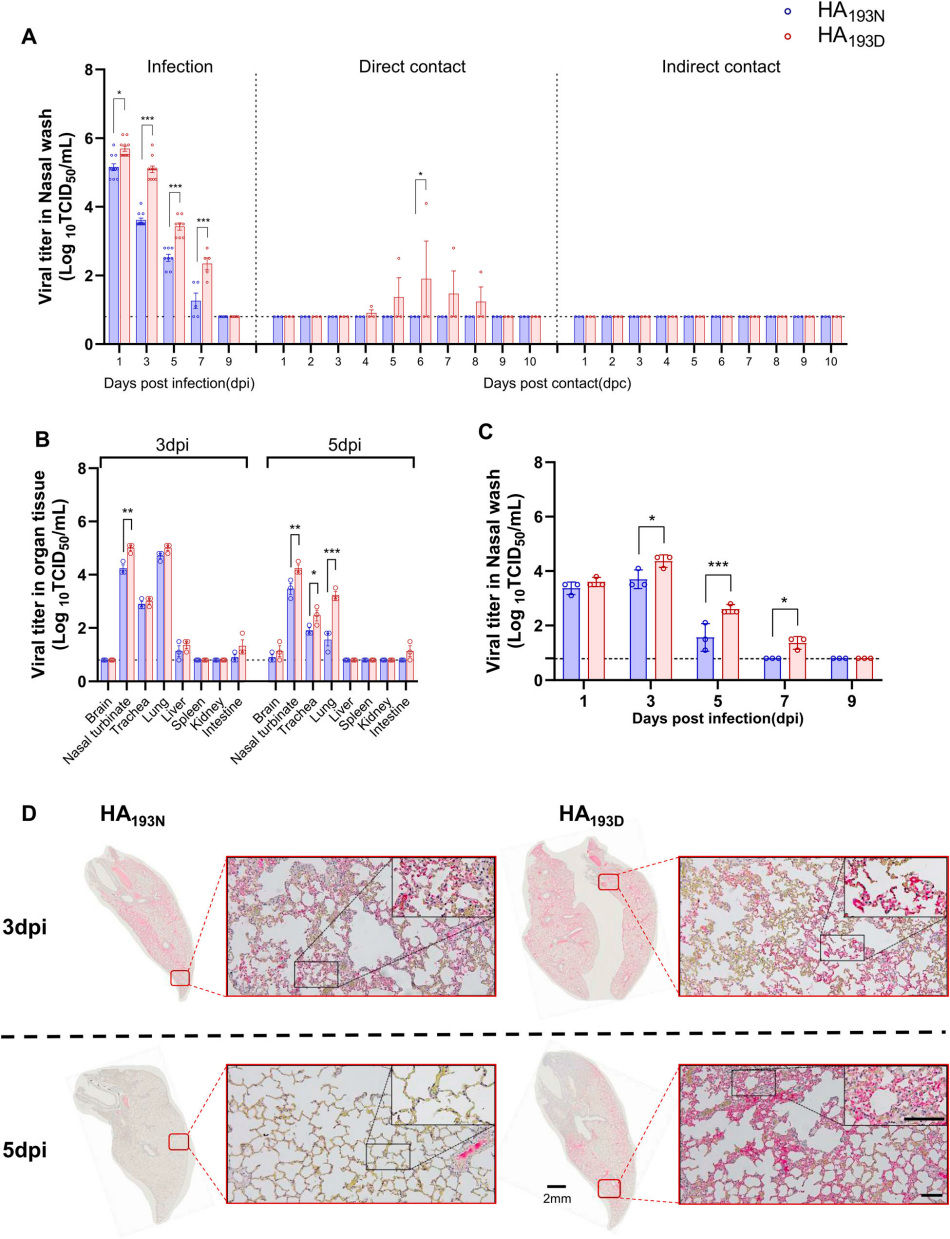
**Comparison of virus replications and transmissions in ferrets**. Groups of ferrets were experimentally inoculation with 10^6.0^ TCID_50_/mL of each designated virus via the intranasal route. To examine transmission, the inoculated animals were individually paired with a direct contact (DC) and an indirect contact (IC) animal (1:1:1 setup, triplicate). Virus titres in the nasal washes (A) and organ tissues (B) of ferrets inoculated with rCT/W811-HA_193N_ and rCT/W811-HA_193D_, respectively. Virus titres in the nasal washes (C) of ferrets low dose (10^4.0^ TCID_50_) inoculated with rCT/W811-HA_193N_ and rCT/W811-HA_193D_, respectively. Detection of influenza nucleoprotein (NP) in the lungs at 3 and 5 dpi with rCT/W811-HA_193N_ and rCT/W811-HA_193D_ viruses in ferrets (C). The limit of virus detection was 0.8 log_10_TCID_50_/mL (dashed lines). Magniﬁcation of 1.0x in the whole lung sections with a 2 mm scale bar. Inserts indicate magnification at 20x and 40x, and the scale bar is 50 µm. The titres shown are means ± SEM from three independently performed experiments. * indicates *p* < 0.05, ** indicates *p* < 0.001, and *** indicates *p* < 0.0001.

Among numerous laboratory animal models, the ferret is considered well qualified for studying both the pathogenicity and transmissibility of human and avian influenza viruses [45]. Thus, to evaluate the zoonotic potentials of rCT/W811-HA_193N_ and rCT/W811-HA_193D_ viruses, influenza A seronegative 16-to 18-week-old female ferrets (n = 9/group) were inoculated intranasally with 10^6.0^ TCID_50_/mL with each of the respective viruses and virus titres monitored in the upper respiratory tract for 9 dpi. All virus-infected ferrets showed marked increases in body temperatures up to 7 dpi, and continuous bodyweight losses were observed up to 10 dpi (Supplementary Figure 3C-F). Further, nasal wash specimens of all infected ferrets were positive at 7 dpi, with the highest titres observed at 1 dpi and gradually decreasing until 7 dpi (Figure 6A, left panel). Interestingly, the rCT/W811-HA_193D_ virus showed much higher virus titres in the nasal washes from 3 to 7 dpi than did the rCT/W811-HA_193N_ virus. To investigate whether each virus could replicate effectively in the ferret upper respiratory tracts, groups of ferrets (n = 3/group) were infected at a lower viral dose (10^4.0^ TCID_50_). Results showed that each virus exhibited a similar replication trend and shedding compared to the high-dose infection, wherein ferrets infected with the rCT/W811-HA_193D_ shed the virus up until 7 dpi with higher viral titres compared to the rCT/W811-HA_193N_ (Figure 6C). To evaluate the tissue distribution of rCT/W811-HA_193N_ and rCT/W811-HA_193D_ viruses in infected ferrets, three animals from each group were sacrificed at 3 and 5 dpi and tissues were collected for virus titration (Figure 6B). Both rCT/W811-HA_193N_ and rCT/W811-HA_193D_ infectious viruses were detected in the nasal turbinates, trachea, lungs, liver, and in intestines at 3 dpi, with the rCT/W811-HA_193D_ virus showing markedly higher virus titres in nasal turbinates compared to the rCT/W811-HA_193N_ virus. Further, although attenuated virus titres were observed at 5 dpi compared to 3 dpi, significantly higher virus titres were detected in the nasal turbinate and intestine of rCT/W811-HA_193D_-infected ferrets compared with the rCT/W811-HA_193N_-infected ferrets, suggesting rCT/W811-HA_193D_ possesses high replication ability in the ferret upper respiratory tract (Figure 6A and B).

To determine the impact of the rCT/W811-HA_193D_ mutation compared to rCT/W811-HA_193N_ on virus transmissibility, three ferrets were added as direct contact (DC) and three as indirect contact (IC) sentinels, as previously described [46]. Although the infectious virus was not recovered from the nasal washes of either IC ferret group (Figure 6A, right panel), the rCT/W811-HA_193D_ virus was isolated in the nasal washes from 1 of the 3 DC ferrets at 4 dpc until 8 dpc, with a peak virus titre of 4.1 log_10_TCID_50_/mL at 6 dpc (Figure 6A, middle panel). Further, the hemagglutination-inhibition (HI) assay revealed that all DC ferrets seroconverted by 10 dpc (20-80 HI units), while no infectious viruses were detected in the nasal wash specimens nor in seroconverted rCT/W811-HA_193N_ DC ferrets (Supplementary Table 3). Collectively, these results suggest that the rCT/W811-HA_193D_ virus could be transmitted to direct contact animals, while rCT/W811-HA_193N_ showed no ferret-to-ferret transmission.

### Competition assay of rCT/W811-HA_193N_ and rCT/W811-HA_193D_ viruses for replication and transmission in infected ferrets

To further investigate the competitive advantage of the N193D substitution within the HA protein, we set up an *in vivo* competition experiment to assess the variation in replication and transmission between rCT/W811-HA_193N_ and rCT/W811-HA_193D_ in ferrets. In a direct one-to-one transmission study, three ferrets were intranasally infected at a 10^6.0^ TCID_50_/mL with a mixture of rCT/W811-HA_193N_ and rCT/W811-HA_193D_ at a 1:1 ratio based on their infectious titres (ratios of each virus was re-confirmed by next-generation sequencing (NGS) at 50% ± 1.7). Infected ferrets showed virus titres as high as ∼5.4 log_10_ viral RNA copies per 0.2 mL at 1 dpi which persisted until 7 dpi, but with a gradually decreasing pattern (Figure 7A). Interestingly, real-time PCR results demonstrated that all three DC ferrets showed 1.0–2.7 log_10_ viral RNA copies per 0.2 mL from the nasal washes suggesting ferret to ferret transmission, although viral RNA loads were relatively limited (Figure 7A). To investigate the virus ratio between rCT/W811-HA_193N_ and rCT/W811-HA_193D_, NGS analysis was performed with each collected nasal wash specimen. The rCT/W811-HA_193D_ strain prevailed over rCT/W811-HA_193N_ in all three directly infected ferrets from 1 dpi (80.81-85.55%) to 7 dpi (99.15-99.84%) (Figure 7B). Surprisingly, only the rCT/W811-HA_193D_ virus was detected in all three direct contact ferrets, wherein DC ferrets from pair 1 and pair 2 shed the virus starting at 4 dpc, while pair 3 exhibited virus transmission starting on day 5 post-contact (Figure 7B). rCT/W811-HA_193N_ was not detected in direct contact ferrets. These results demonstrate that rCT/W811-HA_193D_ readily infects and replicates preferentially in infected ferrets compared to rCT/W811-HA_193N_, and thus rCT/W811-HA_193D_ was the only strain to successfully transmit in contact ferrets.

### Comparison of rCT/W811-HA_193N_ and rCT/W811-HA_193D_ viral infectivity in ALI cell culture of HBEpC

**Figure 8.**
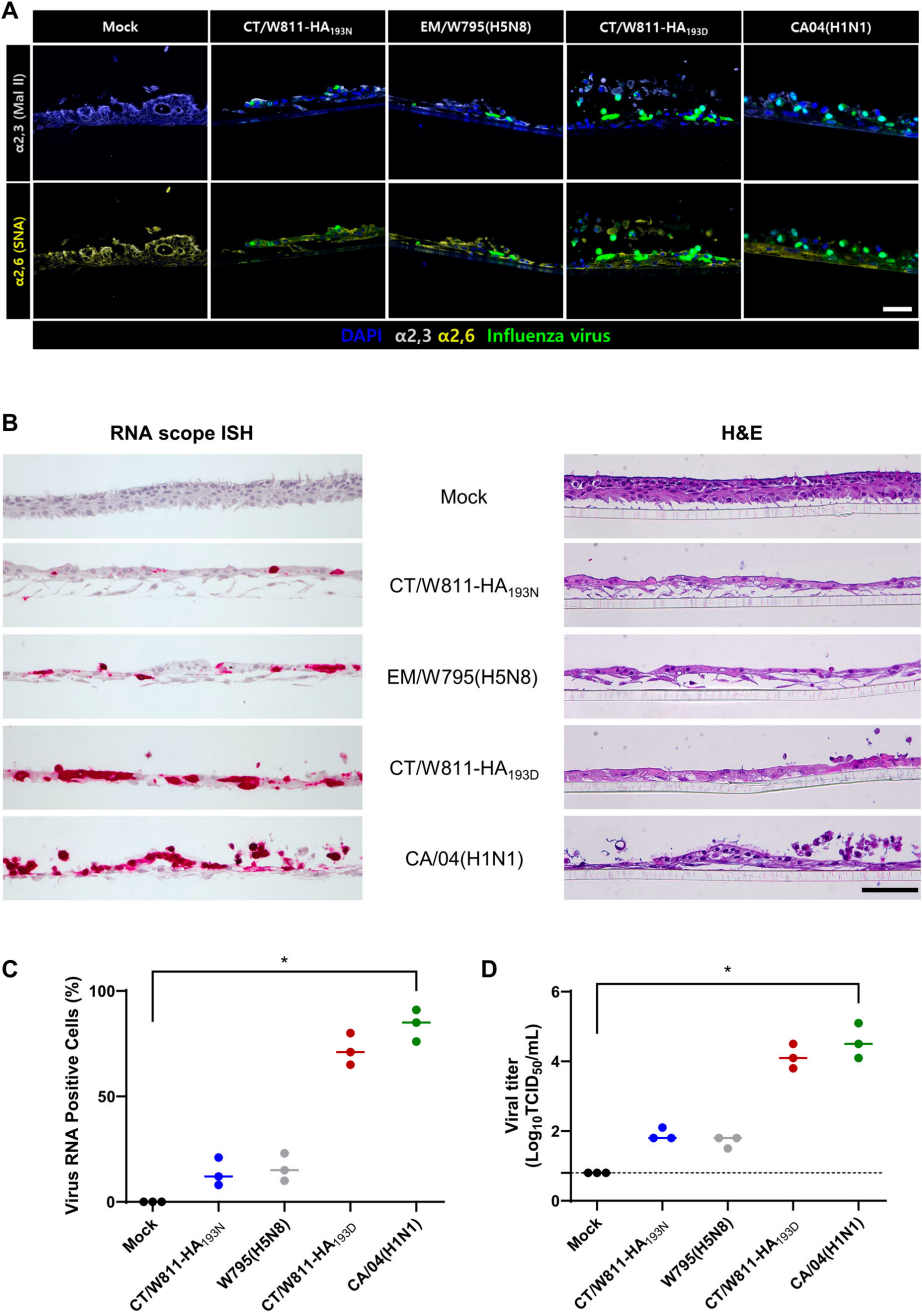
**Immunostaining and RNAscope *in situ* hybridization of influenza NP expression in Air-Liquid Interface (ALI) culture of Human Bronchial Epithelial Cells (HBEpC).** Immunostainings of ALI culture of HBEpC (A). Blue, nucleus (DAPI); green, influenza NP; light grey, Mal II(α2,3); yellow, SNA(α2,6). Total magnification, × 400 for each figure. Scale bar, 50 μm. ALI-HBEpC organoid was infected with each virus and at 24 h post-infection, each infected organoid tissue was fixed, and RNAscope *in situ* hybridization and H&E staining on the consecutive sections of the same organoid sites were performed. RNAscope *in situ* hybridization (B, left panel) and H&E-stained (B, right panel). Scale bar, 50 μm. Influenza viral RNA was detected using an influenza NP probe (Advanced Cell Diagnostics, 504141) and was visualized using RNAscope 2.5 HD Reagent Kit RED. Positive influenza NP cumulative number from influenza NP stained HBEpCs in each slide (400x magnification) and data are presented as mean values ± SEM (n = 3 per virus) (C). Viral titres in ALI cultured NHBE cells 48 h after infection with each virus (D). The limit of virus detection was 0.8 log_10_TCID_50_/mL (dashed lines). * indicates *p* < 0.05.

To provide new insight into human physiology and pathology, organoids have been applied as models of certain human diseases, including infectious diseases. Thus, to evaluate the replication of rCT/W811-HA_193N_ and rCT/W811-HA_193D_ (H5N1) viruses, HBEpC-derived organoid tissues were infected with 0.1 MOI of each virus and the viral antigen and viral RNA were detected by immunostaining and RNAscope *in situ* hybridization, respectively (Figure 8A and B). For comparison, EM/W795(H5N8) and CA/04(H1N1) were used for avian- and human-origin control virus infections, respectively, and compared with PBS mock control organoid block. The avian EM/W795(H5N8) and rCT/W811-HA_193N_ viruses showed relatively minimal infection of α2,3-SAs expression sites, while, the human CA/04(H1N1) virus and the rCT/W811-HA_193D_ virus infected sites expressing α2,6-SAs in the immunostaining data (Figure 8A). The ciliated bronchial epithelial cells were heavily infected by the human CA/04(H1N1) virus and rCT/W811-HA_193D_ virus 24 h after the inoculation causing damage to all of the ciliated epithelium (Figure 8B). However, the avian EM/W795(H5N8) and rCT/W811-HA_193N_ viruses showed only a portion of shortened or destroyed ciliated epithelium as a result of infection. These results were consistent with the immunostaining data (Figure 8B). At 24 hpi, 13.6% and 72% of the HBEpC were infected by rCT/W811-HA_193N_ and rCT/W811-HA_193D_ viruses, respectively (Figure 8C), highlighting differences in infection kinetics between the two viruses. In addition, the rCT/W811-HA_193D_ virus showed higher viral titres than rCT/W811-HA_193N_ in HBEpC, reflecting what was found in MDCK, A549, Calu-3, and NHBE cells (Figure 8D). These results are well within agreement with the receptor binding assay showing that the 193D substitution in the HA receptor binding pocket substantially enhances α2,6-SAs binding affinity, which leads to increased replication in primary human bronchial epithelial cells.

### Stability of the HA-193 mutation

Given the dynamic viral adaptations or reverse mutations that can occur during virus infections [47], next-generation sequencing was performed on the viruses to evaluate the stability of mutations in the HA-193. Briefly, cell infection supernatants were collected at different time points (12 hpi, 24 hpi, and 48 hpi), results indicated that the mutation, either HA_193D_ or HA_193N_, was consistently maintained among the cell lines infected with each virus (Supplementary Figure 4A). Similarly, we investigated the potential occurrence of HA-193 reverse mutations or adaptive mutation in the in vivo experiments. NGS analyses on oropharyngeal swabs, mice lungs, and ferret nasal wash samples collected at different time points were conducted. While no reverse mutations were detected in the mice (Supplementary Figure 4B), interestingly, in chickens, dynamic viral adaptation was observed in the oropharyngeal swabs collected at 3 dpi, specifically in chickens infected with rCT/W811-HA_193D_, wherein a reverse mutation to HA_193N_ was detected at a frequency of >60% (Supplementary Figure 4B). Additionally, ferrets inoculated with rCT/W811-HA_193N_ exhibited a gradual and continuous mutation to HA_193D_, with the HA_193N_ virus gradually decreasing in nasal washes as HA_193D_ predominated (Supplementary Figure 4B). Taken together, findings suggest that both HA-193N and HA-193D mutations are stable in cell culture conditions across avian and mammalian origins. Notably, the distinct characteristics observed in in vivo studies indicate host factors may contribute to adaptation or reverse mutation.

## Discussion

HPAI H5Nx outbreaks occur continually worldwide and have become a major threat to animal and human public health. Of the many clades of HPAI H5 viruses, clade 2.3.4.4b HPAI H5Nx viruses had been the main cause of outbreaks among Asia and European countries [48]; however, since October 2021, reassortant clade 2.3.4.4b H5N1 viruses have been the predominating strain of avian influenza globally [49]. The introduction and spread of this virus through migratory birds is unavoidable, as periodically there are huge outbreaks in domestic poultry due to the interaction of migratory birds with terrestrial poultry, which has occasionally resulted in human infections [50]. Therefore, continuous surveillance of HPAI H5 viruses in wild migratory bird habitats and prompt analysis can provide an early warning for any potential threat to humans caused by deadly avian influenza viruses.

In this regard, we characterized the HPAI 2.3.4.4b H5N1 viruses isolated from wild birds in South Korea during the 2021–2022 winter season. All 2021–22 H5N1 isolates share similar genetic markers of pathogenic phenotypes, interestingly, we found an aspartic acid residue substitution at position 193 (193D) of the receptor-binding domain (RBD) in two out of six isolates. While most avian influenza viruses have an Asn (N), Arg (R), or a Lys (K) at residue 193, a recent study showed that the K193T mutation in the RBS of avian H5 viruses could confer human-type receptor specificity [24], suggesting a critical role of residue 193 in the RBD of HA protein in receptor binding specificity. Therefore, to better understand the impact of the N193D mutation, rCT/W811-HA_193N_ and rCT/W811-HA_193D_ viruses were generated using reverse genetics, and their receptor binding specificity, replication, and virulence were evaluated in three animal models. Interestingly, rCT/W811-HA_193D_ showed increased mammalian (SAα2,6Gal) receptor binding affinity compared to rCT/W811-HA_193N._ Further, rCT/W811-HA_193N_ exhibited more rapid death in infected chickens compared with rCT/W811-HA_193D_ viruses, while the rCT/W811-HA_193D_ virus was found to be more pathogenic in mice than the rCT/W811-HA_193N_ virus, with an MLD_50_ of 10^2.7^ vs. 10^3.8^ EID_50_/mL, respectively. Notably, BALB/c mice employed in this study expresses both avian and mammalian receptors [51], the rCT/W811-HA_193D_ was demonstrated to have a dual binding activity in which the virus can utilize both receptors during infection, leading to a higher virus replication, severe disease phenotype, and lethality. In ferret infection studies, rCT/W811-HA_193D_ showed significantly higher virus replication in the upper respiratory tract and exhibited multi-systemic viral replication compared to rCT/W811-HA_193N_. While historically avian HPAI H5N1 viruses exhibit limited ferret-to-ferret transmissibility, all direct contact ferrets of rCT/W811-HA_193D_ were seroconverted at 10 dpc, but none of the rCT/W811-HA_193N_ direct contact ferrets seroconverted. These results suggest that naturally occurring rCT/W811-HA_193D_-like viruses may pose a potential risk for human infection. Several studies have reported that altering receptor specificity, specifically shifting from avian (SAα2,3Gal) to human (SAα2,6Gal) receptors, plays a pivotal role in facilitating the cross-species transmission of influenza A viruses. In fact, Yamada et. al, demonstrated mutations among H5 AIVs that cause similar receptor affinity switches, particularly mutations at residues 186 and 196 of HA protein RBD (H3 numbering) [39]. Further, Wan et. al also reported increased variation at position 193 of the HA 190-helix among H9N2 viruses in China since 2013 [27] and demonstrated that mutations of the 193E residue significantly impact virus replicative fitness, receptor binding, and *in vivo* transmissibility in chickens [52]. Further, it is noteworthy that the H7N9 viruses isolated during the first wave of infection in China in 2013 maintained significant binding affinity for the avian (SAα2,3Gal) receptor, but the continuous circulation of H7N9 until 5th waves lead to more amino acid substitutions within the RBD, resulting in a complete switch to SAα2,6Gal specificity [23]. Similarly, our competition experiment demonstrated that when equal amounts of virus were used to infect ferrets, the rCT/W811-HA_193D_ virus exhibited increased replication, becoming more than 90% dominant as early as 3 dpi demonstrating replication advantages in the upper airway of ferrets. Eventually, only rCT/W811-HA_193D_ could transmit to naïve contact animals in these studies. These results suggest that even though several variants may circulate in nature, strains with enhanced replicative fitness advantages could be selected in a novel host leading to competitive transmission advantages.

The global spread of novel H5Nx viruses in avian species is inevitable, and will likely generate more complicated gene combinations following multiple reassortment events. While currently, the avian influenza H5N1 outbreak situation is primarily an animal health issue, human infection cases of clade 2.3.4.4b H5Nx viruses have been sporadically detected in Asia, Europe, and the United States [53]. Moreover, the recent report of human infection with clade 2.3.4.4b HPAI H5N8 in Russia in 2020 suggests an increasing ability of these viruses to cross species barriers [54]. Further, sequence analysis of contemporary HPAI H5Nx viruses isolated from humans revealed that the HA-193D mutation is present in 12.9% of H5N6 human isolates. Herein, we demonstrated that although both HA_193D_ and HA_193N_ viruses are HPAI as shown in the chicken studies, results of the mice and ferret studies clearly show differences in infection characteristics imposed by the HA-193 mutation. This mutation may be one of the critical amino acid changes which could result in strong mammalian adaptability among emerging novel HPAI H5Nx viruses and the presence of such mutation implies a higher risk of avian-to-human infection from these viruses.

In this study, we confirmed the ability of the rCT/W811-HA_193D_ to replicate efficiently in ferrets and human bronchial epithelial (HBE) cells, showing replication efficiency similar to that of the 2009 pandemic CA/04 H1N1 influenza virus, suggesting that these viruses pose a potential threat to human infection. Although detailed pathogenicity tests may not be available for each novel H5Nx outbreak, it is imperative to conduct a thorough risk assessment for emerging/mutant viruses with known virulence factors and evolutionary changes in host susceptibility. Furthermore, considering the massive outbreaks of HPAI H5N1 viruses in wild birds and domestic poultry, more precise, broad-reaching molecular studies are required to determine whether these viruses evolve into novel subtypes or genotypes as a result of reassortment events in wild and domestic birds. Therefore, this work re-emphasizes the significance of active and continuous surveillance of avian influenza viruses in wild birds and poultry, as along with the virulence characterization through animal experiments in mammals.

## Author contributions

S.G.J., Y.I.K., and Y.K.C. designed research; S.G.J., Y.I.K., M.A.B.C., J.H.C., J.R.G., R.R., E.H.K., S.M.K., M.S.S., and Y.K.C. designed and performed the animal study; S.G.J., Y.I.K., H.Y.J., and D.B.P. analyzed data; J.H., J.W.A., and M.H.K. docking studies; Y.I.K., M.A.B.C., and Y.K.C wrote the paper.

## Supplementary Material

Supplementary_materials_final_revised
