# Supplementary material for: HA N193D substitution in the HPAI H5N1 virus alters receptor binding affinity and enhances virulence in mammalian hosts
Source: Emerg Microbes Infect. 2024 Jan 8;13(1):2302854. doi: 10.1080/22221751.2024.2302854 (PMC10840603; doi:10.1080/22221751.2024.2302854)
Supplement: Supplementary_materials_final_revised [file TEMI_A_2302854_SM6779.pdf]

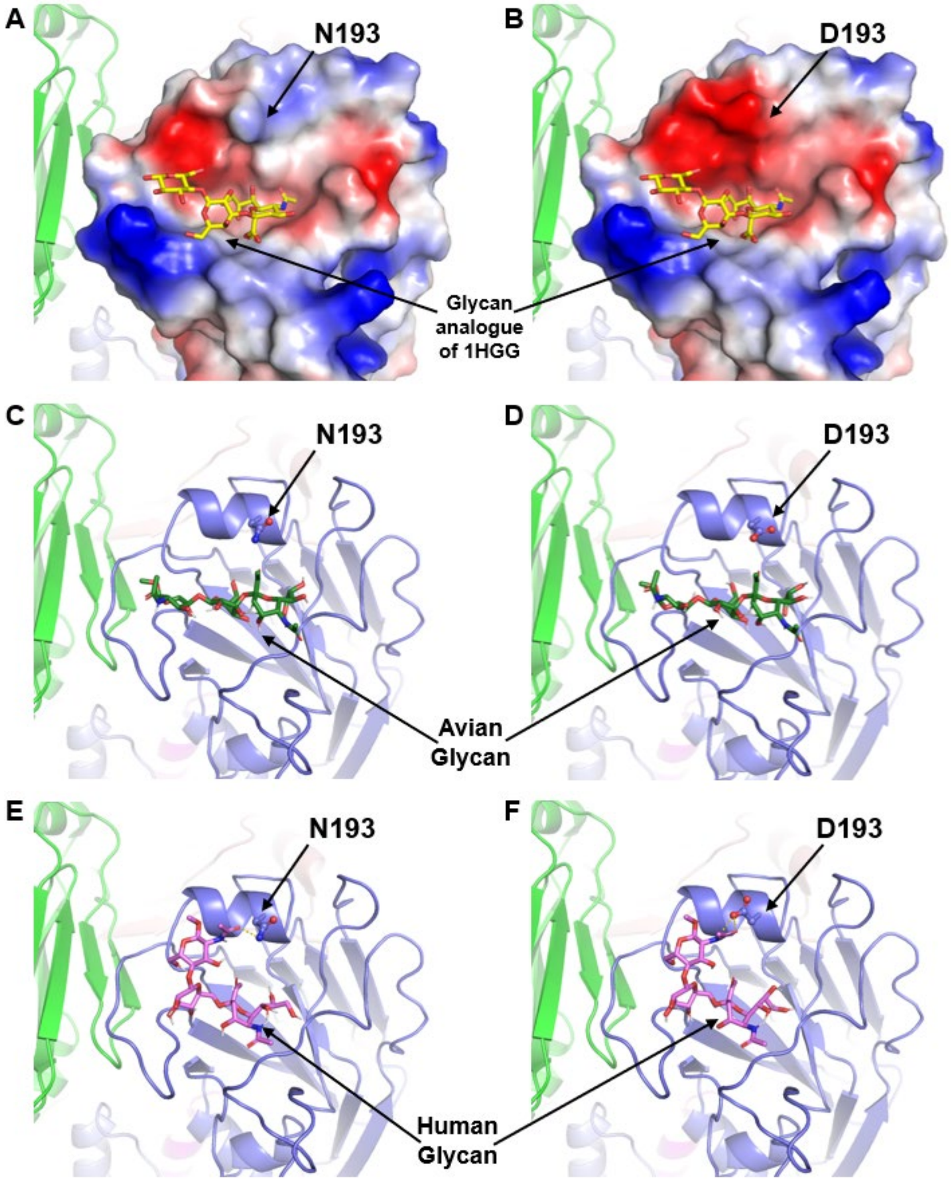

Supplementary Figure 1. Docking prediction for interactions between HA and glycans. Docking prediction for interactions between HA and glycans. Electrostatic surface of the sialic binding site in HA-193N (A) and HA-193D (B). The electrostatic surface was prepared from the protein structure of H5N1 influenza virus hemagglutinin after the substitution of HA-193K using Pymol software (Schrödinger). A glycan analogue (yellow-stick) is placed in superposition to the hemagglutinin structure containing an analog (PDB code: 1HGG). The docking simulations for avian glycans with HA-193N (C) and HA-193D (D) are depicted. HA protein and RBS are drawn as blue-purple cartoons, and the ligands (avian glycan) are displayed in dark-green sticks. For human glycans, docking simulations with HA-193N (E) and HA-193D (F) are shown. In these simulations, the ligands (human glycans) are colored magenta. The areas of hydrophilic interaction are highlighted with yellow dotted lines.

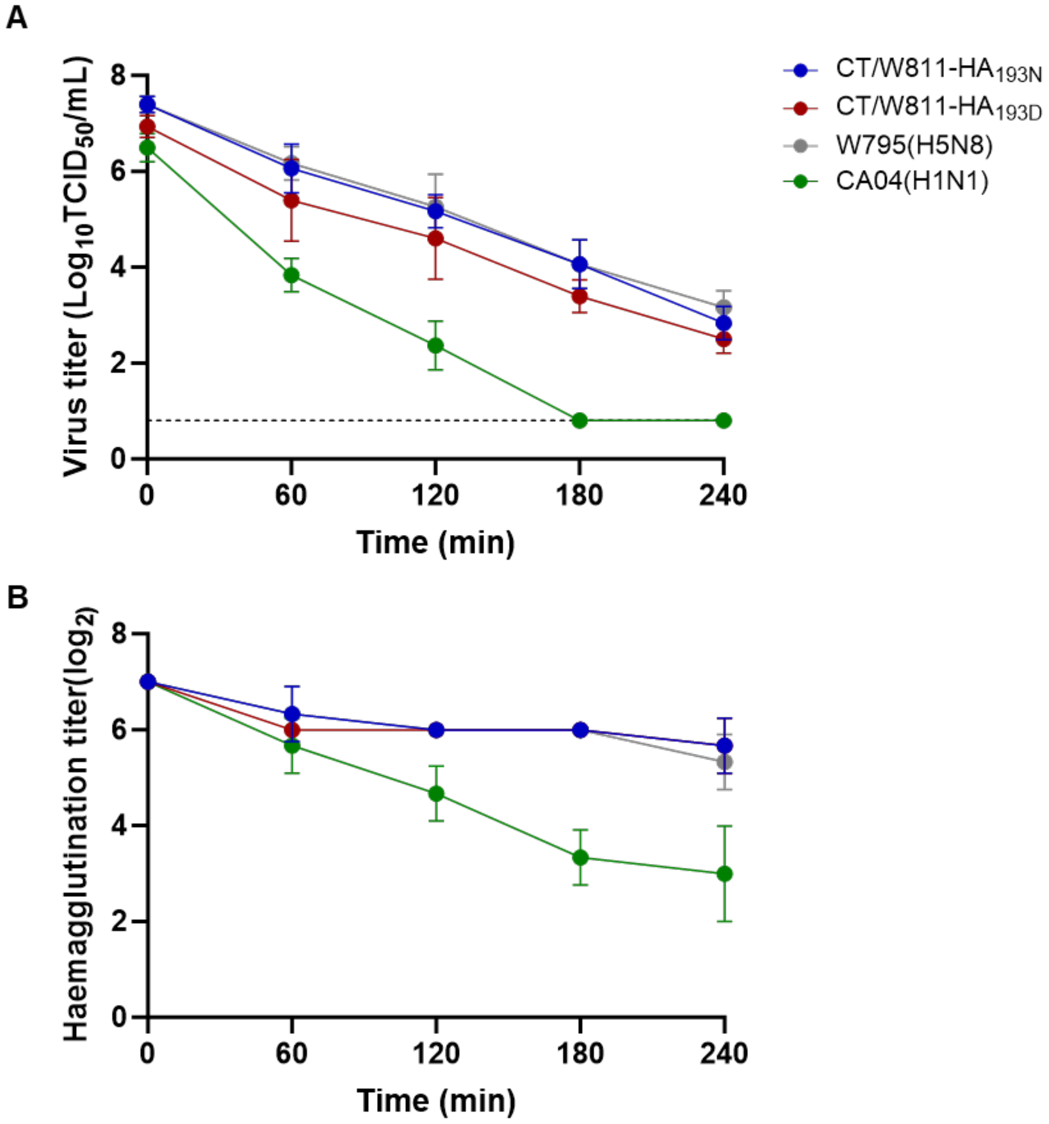

Supplementary Figure 2. Impact of thermal processing on viral infectivity and hemagglutination. A virus stock comprising 128 HA units was used to incubate aliquots at 50 °C for the specified times. Using $TCID_{50}$ assays on MDCK cells, the virus loads in heat-treated samples were quantified (A). Hemagglutination titres in heat-treated samples were determined by using hemagglutination assays with 0.5% TRBCs (B). Each point represents the mean ± standard deviation from triplicate experiments.

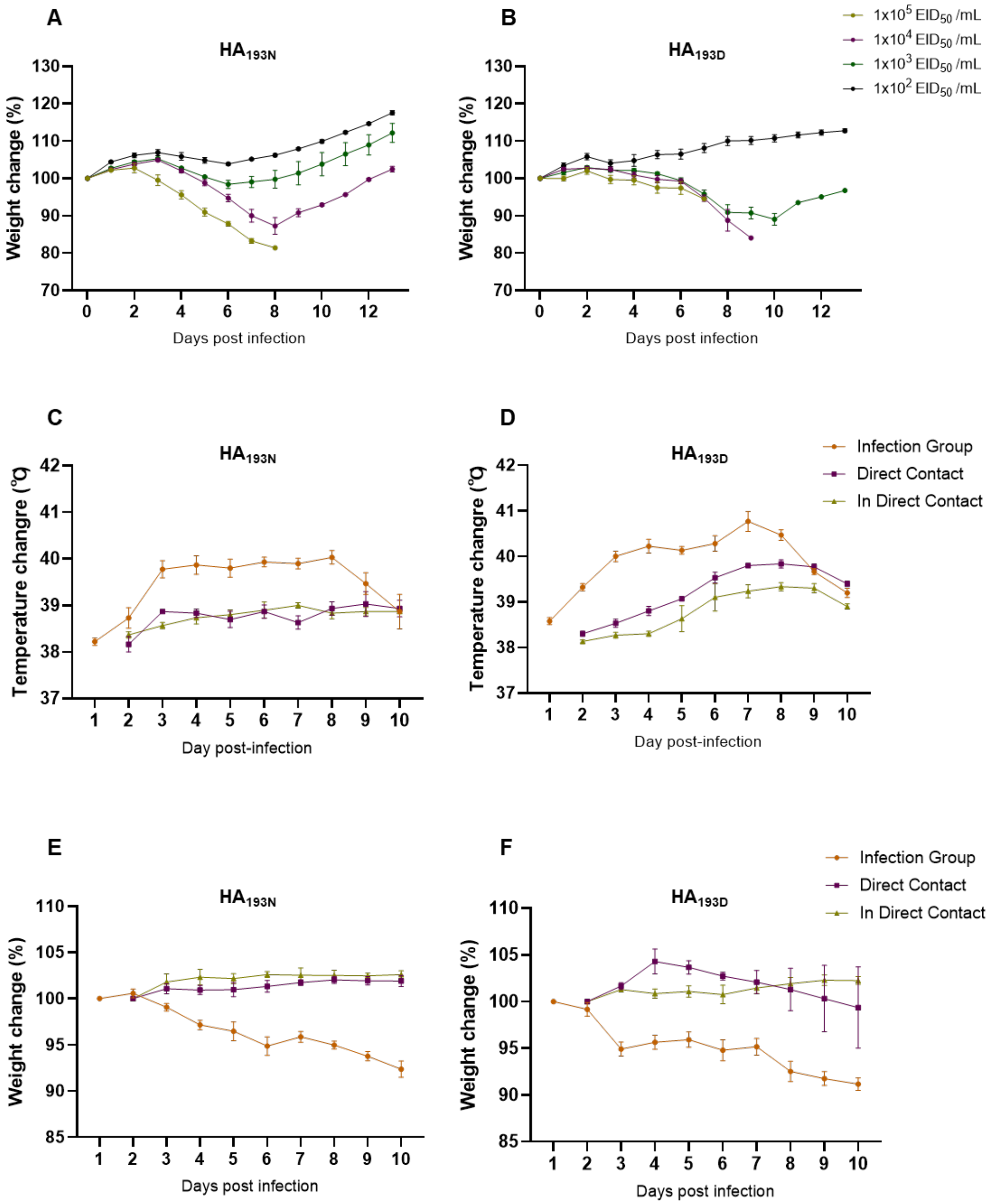

Supplementary Figure 3. Body weight loss and body temperature changes after CT/W811-HA193N and CT/W811-HA193D challenge. Groups of female BALB/c (n= 5/ group) mice were intranasally infected with the CT/W811-HA193N or CT/W811-HA193D at 10^5, 10^4, 10^3, or 10^2 EID_{50}/mL, respectively. The CT/W811-HA193N infected groups body weight changes (A) and the CT/W811-HA193D infected groups body weight changes (B). Temperature changes in the CT/W811-HA193N infected group (C) and CT/W811-HA193D infected group (D), and body weight changes in the CT/W811-HA193N infected group (E) and CT/W811-HA193D infected group (F) in ferret infection experiment of two viruses. Data are presented as mean value ± SEM.

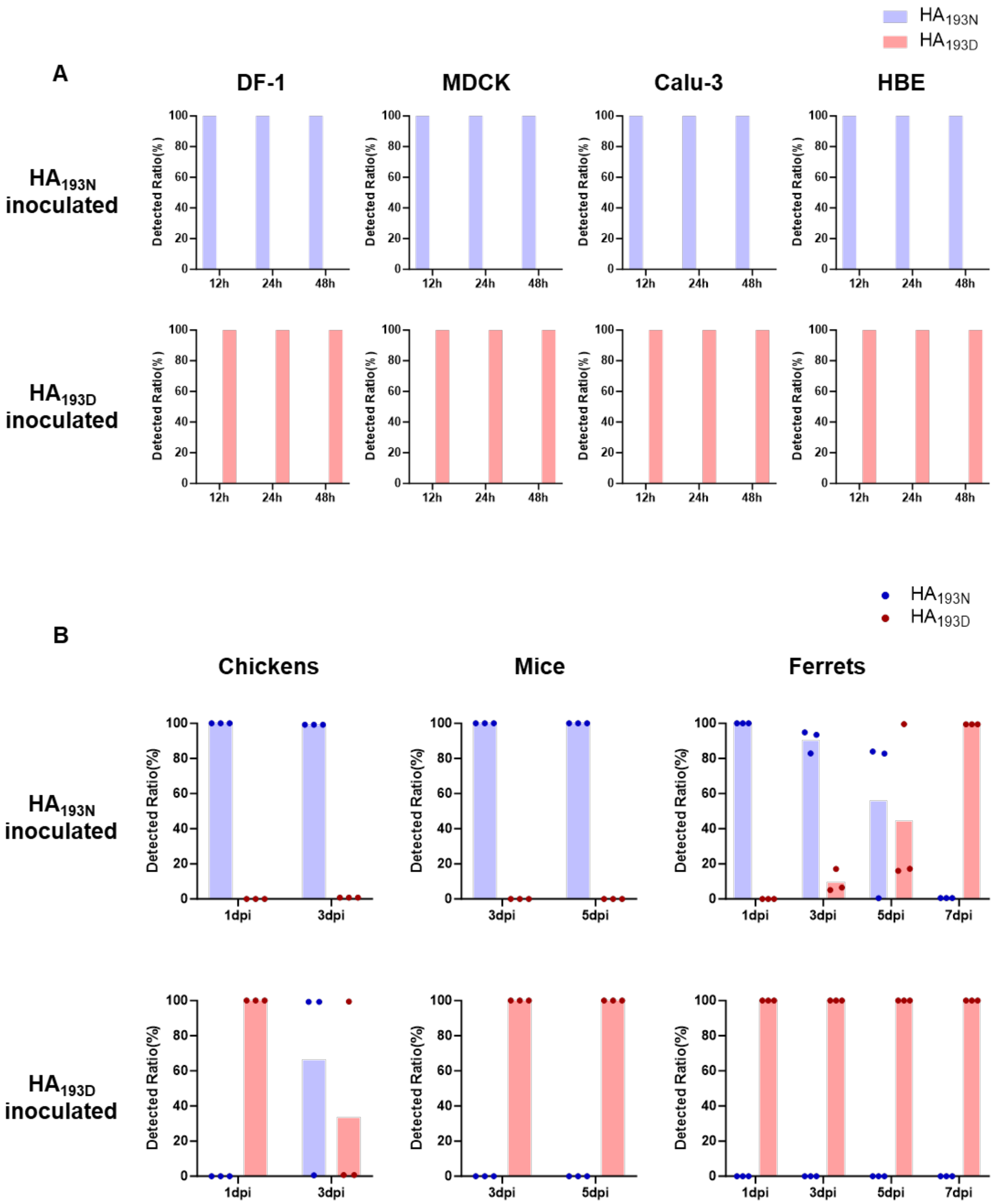

Supplementary Figure 4. Progressive sequence changes of the virus as it passes through *in vitro* and *in vivo*. Dynamic mutation frequencies of different amino acids at HA-193 during replication of CT/W811-HA_{193N} and CT/W811$_{193D}$ viruses in cell lines (A), and animals (B). Each dot represents the proportion detected in an individual animal, and the columns are the average frequencies.

Supplementary Table 1. Molecular analysis of previously isolated H5 HPAI viruses and the 2021/22 Korean H5 HPAI viruses.

Viruses[†]	HA clade	HA sequence (aa)									HA deletion	NA stalk deletion	NS1			PB2 sequence at Aa		Expression of PB1-F2 protein
		Cleavage site	Receptor Binding Sites										Deletion of Aa 80-84	Aa residue at				
		335-348[a]	158	193	222	224	226	227	228	318	133	49-68		92	C-term	627	701	
**A/CT/Korea/W811/21 (H5N1)**	**2.3.4.4**	**RERRRKR/G**	**N**	**D**	**Q**	**N**	**Q**	**R**	**G**	**T**	**NO**	**NO**	**NO**	**D**	**ESEV**	**E**	**D**	**YES**
**A/CT/Korea/W812/21 (H5N1)**	**2.3.4.4**	**RERRRKR/G**	**N**	**N**	**Q**	**N**	**Q**	**R**	**G**	**T**	**NO**	**NO**	**NO**	**D**	**ESEV**	**E**	**D**	**YES**
**A/CT/Korea/W813/21 (H5N1)**	**2.3.4.4**	**RERRRKR/G**	**N**	**D**	**Q**	**N**	**Q**	**R**	**G**	**T**	**NO**	**NO**	**NO**	**D**	**ESEV**	**E**	**D**	**YES**
**A/CT/Korea/W814/21 (H5N1)**	**2.3.4.4**	**RERRRKR/G**	**N**	**N**	**Q**	**N**	**Q**	**R**	**G**	**T**	**NO**	**NO**	**NO**	**D**	**ESEV**	**E**	**D**	**YES**
**A/CT/Korea/W815/21 (H5N1)**	**2.3.4.4**	**RERRRKR/G**	**N**	**N**	**Q**	**N**	**Q**	**R**	**G**	**T**	**NO**	**NO**	**NO**	**D**	**ESEV**	**E**	**D**	**YES**
**A/CT/Korea/W816/21 (H5N1)**	**2.3.4.4**	**RERRRKR/G**	**N**	**N**	**Q**	**N**	**Q**	**R**	**G**	**T**	**NO**	**NO**	**NO**	**D**	**ESEV**	**E**	**D**	**YES**
A/MD/Korea/WA585/21 (H5N1)	2.3.4.4	REKRRKR/G	N	N	Q	N	Q	R	G	T	NO	NO	NO	D	ESEV	E	D	YES
A/EM/Korea/W795/20 (H5N8)	2.3.4.4	REKRRKR/G	N	N	Q	N	Q	R	G	T	NO	NO	NO	D	GSEV	E	D	YES
A/MD/Korea/W452/14 (H5N8)	2.3.4.4	RERRRKR/G	N	N	Q	N	Q	R	G	T	NO	NO	NO	D	ESEVRG	E	D	YES
A/Bdk/Korea/Gochang1/14 (H5N8)	2.3.4.4	REKRRKR/G	N	N	Q	N	Q	R	G	T	NO	NO	NO	D	ESEV	E	D	YES
A/EM/Korea/W149/06 (H5N1)	2.2	GERRRKKR/G	N	K	K	N	Q	S	G	T	NO	YES	YES	D	ESKV	K	D	YES

Aa, amino acid; RBS, receptor binding site; C-term, 4 amino acid sequence at the C-terminal end.

AP; Anas platyrhynchos, BDk; breeder duck, CT; common teal, EM; environment, MD; mallard duck.

[†]The isolates in boldface are the 2021 Korean HPAI H5N1 viruses examined in this study.

[a]H3 numbering.

Supplementary Table 2. The intravenous pathogenicity index (IVPI) test result of CT/W811-HA193N and CT/W811-HA193D viruses.

		Day 1	Day 2	Day 3	Day 4	Day 5	Day 6	Day 7	Day 8	Day 9	Day 10	Total	Weight	SUM
**CT/W811-HA$_{193N}$**	**Normal**	0	0	0	0	0	0	0	0	0	0	0	0	0
	**Sick**	0	0	0	0	0	0	0	0	0	0	2	1	2
	**Severe Sick**	7	0	0	0	0	0	0	0	0	0	7	2	14
	**Dead**	3	10	10	10	10	10	10	10	10	10	93	3	279
											Total Recordings	100		293[a]
**CT/W811-HA$_{193D}$**	**Normal**	0	0	0	0	0	0	0	0	0	0	0	0	0
	**Sick**	2	0	0	0	0	0	0	0	0	0	2	1	2
	**Severe Sick**	6	0	0	0	0	0	0	0	0	0	6	2	12
	**Dead**	2	10	10	10	10	10	10	10	10	10	92	3	276
											Total Recordings	100		290[b]

[a]The IVPI value of the CT/W811-HA$_{193N}$ strain is 2.93.
[b]The IVPI value of the CT/W811-HA$_{193D}$ strain is 2.90.

Supplementary Table 3. Seroconversion of the ferrets in CT/W811-HA$_{193N}$ and CT/W811-HA$_{193D}$ viruses transmission studies.

Virus	Seroconversion: positive/total (HI titers)[a]			
	Inoculated		Direct contact (DC)	Indirect contact (IC)
	CT/W811-HA$_{193N}$	CT/W811-HA$_{193D}$		
CT/W811-HA$_{193N}$	3/3 (160, 160, 80)	3/3 (160, 160, 320)	0/3	0/3
CT/W811-HA$_{193D}$	3/3 (160, 320, 160)	3/3 (160, 320, 320)	3/3 (20, 20, 80)	0/3

[a]Sera were collected from ferrets on day 11 dpi (10dpc). Seroconversion was confirmed by hemagglutination inhibition (HI) assay

Supplementary Table 4. GISAID accession numbers of H5-HA_{193D} viruses used in the study

Virus name	subtype	segment	Accession Number
A/Common Teal/South Korea/W811/2021	H5N1	Hemagglutinin(HA)	EPI1950415
A/Common Teal/South Korea/W812/2021	H5N1	Hemagglutinin(HA)	EPI1950423
A/Common Teal/South Korea/W813/2021	H5N1	Hemagglutinin(HA)	EPI1950431
A/Common Teal/South Korea/W814/2021	H5N1	Hemagglutinin(HA)	EPI1950439
A/Common Teal/South Korea/W815/2021	H5N1	Hemagglutinin(HA)	EPI1950447
A/Common Teal/South Korea/W816/2021	H5N1	Hemagglutinin(HA)	EPI1950455
A/Chicken/Hunan/176/2010	H5N1	Hemagglutinin(HA)	EPI1892898
A/Duck/Saratov/29-11V/2021	H5N1	Hemagglutinin(HA)	EPI1922973
A/Eurasian Wigeon/Netherlands/1/2020	H5N1	Hemagglutinin(HA)	EPI1807243
A/Teal/Miyazaki/211109-32/2021	H5N1	Hemagglutinin(HA)	EPI2200549
A/Quail/Korea/H526/2021	H5N1	Hemagglutinin(HA)	EPI1938272
A/Peregrine falcon/Kanagawa/1409C001T1/2022	H5N1	Hemagglutinin(HA)	EPI2223226
A/Eurasian wigeon/Hokkaido/M184/2022	H5N1	Hemagglutinin(HA)	EPI2213709
A/Eurasian wigeon/Hokkaido/Q71/2022	H5N1	Hemagglutinin(HA)	EPI2197776
A/Mandarin duck/Korea/WA585/2021	H5N1	Hemagglutinin(HA)	EPI1938252
A/Duck/Guangdong/S4518/2021	H5N1	Hemagglutinin(HA)	EPI2029834
A/Duck/Guangdong/S4525/2021	H5N1	Hemagglutinin(HA)	EPI2029842
A/Chicken/Kagoshima/21A6T/2021	H5N1	Hemagglutinin(HA)	EPI1933663
A/Chicken/Ehime/TU11-2-24,25/2022	H5N1	Hemagglutinin(HA)	EPI2181547
A/Chicken/Ehime/TU10-2-13/2022	H5N1	Hemagglutinin(HA)	EPI2181515
A/Chicken/Ehime/TU12-2-16,17/2022	H5N1	Hemagglutinin(HA)	EPI2200557
A/Chicken/Japan/AQ-HE30-35C1/2018	H5N2	Hemagglutinin(HA)	EPI1776388
A/Chicken/Fujian/3.15_FZHX0009-C/2018	H5N2	Hemagglutinin(HA)	EPI1820568
A/Chicken/Anhui/2.22_YHZGS007-O/2019	H5N2	Hemagglutinin(HA)	EPI1821250
A/Chicken/Sichuan/J1/2014	H5N6	Hemagglutinin(HA)	EPI675770
A/Sichuan/26221/2014	H5N6	Hemagglutinin(HA)	EPI533583
A/Duck/Hunan/01.21 YYFQH018-O/2015	H5N6	Hemagglutinin(HA)	EPI658855
A/Duck/Hunan/01.21 YYFQH030-O/2015	H5N6	Hemagglutinin(HA)	EPI658975
A/Duck/Hunan/01.21 YYFQH009-O/2015	H5N6	Hemagglutinin(HA)	EPI659079

A/Duck/Hunan/01.21_YYFQH024-P/2015	H5N6	Hemagglutinin(HA)	EPI658935
A/Anser fabalis/Hunan/03.09 YYDTH0007/2015	H5N6	Hemagglutinin(HA)	EPI659295
A/Duck/Hunan/04.14 YYGK460-O/2015	H5N6	Hemagglutinin(HA)	EPI659367
A/Duck/Hunan/04.14 YYGK451-1-O/2015	H5N6	Hemagglutinin(HA)	EPI664597
A/Duck/Hunan/04.14 YYGK0878-P/2015	H5N6	Hemagglutinin(HA)	EPI659519
A/Duck/Hunan/04.14 YYXS877-P/2015	H5N6	Hemagglutinin(HA)	EPI659511
A/Duck/Hubei/03.06 WHWTZ0158-P/2015	H5N6	Hemagglutinin(HA)	EPI658647
A/Duck/Hubei/03.06 WHWTZ0130-O/2015	H5N6	Hemagglutinin(HA)	EPI658663
A/Duck/Hubei/03.06 WHWTZ0123-O/2015	H5N6	Hemagglutinin(HA)	EPI658623
A/Duck/Hubei/03.06 WHWTZ0125-O/2015	H5N6	Hemagglutinin(HA)	EPI658655
A/Duck/Guangxi/04.10 JX046/2015	H5N6	Hemagglutinin(HA)	EPI661703
A/Duck/Hubei/03.06 WHWTZ0129-P/2015	H5N6	Hemagglutinin(HA)	EPI658535
A/Duck/Hubei/03.06 WHWTZ0150-P/2015	H5N6	Hemagglutinin(HA)	EPI664533
A/Duck/Hubei/03.06 WHWTZ0111-O/2015	H5N6	Hemagglutinin(HA)	EPI664525
A/Duck/Hubei/03.06 WHWTZ0124-O/2015	H5N6	Hemagglutinin(HA)	EPI658639
A/Duck/Zhejiang/S4854/2021	H5N6	Hemagglutinin(HA)	EPI1997226
A/Green pheasant/Hunan/10/2015	H5N6	Hemagglutinin(HA)	EPI1639879
A/Peacock/Hunan/15/2015	H5N6	Hemagglutinin(HA)	EPI1639911
A/Ostrich/Guangxi/GX-1/2017	H5N6	Hemagglutinin(HA)	EPI1093302
A/Guangxi/55726/2016	H5N6	Hemagglutinin(HA)	EPI873669
A/Hunan/55555/2016	H5N6	Hemagglutinin(HA)	EPI873668
A/Quail/Zhanjiang/16887/2016	H5N6	Hemagglutinin(HA)	EPI1060753
A/Chicken/Wuxi/7765/2016	H5N6	Hemagglutinin(HA)	EPI1055368
A/Chicken/Ha Tinh/73VTC/2017	H5N6	Hemagglutinin(HA)	EPI1584022
A/Chicken/Dong Nai/25437VTC/2019	H5N6	Hemagglutinin(HA)	EPI1665384
A/Chicken/Ba Ria-Vung Tau/21078VTC/2019	H5N6	Hemagglutinin(HA)	EPI1665376
A/Chicken/Shenzhen/1061/2013	H5N6	Hemagglutinin(HA)	EPI602484
A/Duck/Hunan/12.07 YYGK112-P/2013	H5N6	Hemagglutinin(HA)	EPI656650
A/Environment/Jiangxi/25004/2014	H5N6	Hemagglutinin(HA)	EPI1435225
A/Shenzhen/1/2016	H5N6	Hemagglutinin(HA)	EPI687704
A/Environment/Guangdong/40113/2015	H5N6	Hemagglutinin(HA)	EPI749721
A/Environment/Jiangxi/50449/2016	H5N6	Hemagglutinin(HA)	EPI1435129

A/Chicken/Hunan/04.14 YYGK607-P/2015	H5N6	Hemagglutinin(HA)	EPI659455
A/Chicken/Yunnan/03.15 DQJT0054-Z-P/2015	H5N6	Hemagglutinin(HA)	EPI659095
A/Duck/Vietnam/LBM760/2014	H5N6	Hemagglutinin(HA)	EPI596622
A/Duck/Guangzhou/41227/2014	H5N6	Hemagglutinin(HA)	EPI593896
A/Feline/Guangdong/2/2015	H5N6	Hemagglutinin(HA)	EPI760097
A/Environment/Fujian/28686/2016	H5N6	Hemagglutinin(HA)	EPI1333910
A/Egret/Zhejiang/W15/2017	H5N6	Hemagglutinin(HA)	EPI1901854
A/Duck/Hyogo/1/2016	H5N6	Hemagglutinin(HA)	EPI866708
A/Duck/Jiangxi/01.14 NCJD034-P/2015	H5N6	Hemagglutinin(HA)	EPI664221
A/Duck/Jiangxi/01.14 NCJD064-P/2015	H5N6	Hemagglutinin(HA)	EPI661071
A/Duck/China/FJ18252/2019	H5N6	Hemagglutinin(HA)	EPI1931965
A/Duck/China/FJ18299/2018	H5N6	Hemagglutinin(HA)	EPI1931969
A/Duck/Guangdong/PO17281388/MZH/2017	H5N6	Hemagglutinin(HA)	EPI1366509
A/Duck/China/FJ18248/2019	H5N6	Hemagglutinin(HA)	EPI1931964
A/Duck/China/FJ1914/2019	H5N6	Hemagglutinin(HA)	EPI1931972
A/Duck/China/FJ1829/2019	H5N6	Hemagglutinin(HA)	EPI1931968
A/Duck/China/FJ19332/2019	H5N6	Hemagglutinin(HA)	EPI1931979
A/Duck/China/FJ19323/2019	H5N6	Hemagglutinin(HA)	EPI1931978
A/Anhui/2021-00011/2020	H5N6	Hemagglutinin(HA)	EPI1848299
A/Muscovy duck/China/FJFZ21/2020	H5N6	Hemagglutinin(HA)	EPI1841913
A/Chicken/Suzhou/j6/2019	H5N6	Hemagglutinin(HA)	EPI1429237
A/Chicken/Anhui/2.22_YHZGS003-O/2019	H5N6	Hemagglutinin(HA)	EPI1821248
A/Guangxi/13486/2017	H5N6	Hemagglutinin(HA)	EPI1352861
A/Jiangsu/1/2020	H5N6	Hemagglutinin(HA)	EPI1838640
A/Jiangsu/32888/2018	H5N6	Hemagglutinin(HA)	EPI1352821
A/Duck/Guangdong/PO17281256/MZH/2017	H5N6	Hemagglutinin(HA)	EPI1366506
A/Duck/Guangdong/G1378/2018	H5N6	Hemagglutinin(HA)	EPI1255953
A/Goose/Guangdong/7.20_DGCP010-C/2017	H5N6	Hemagglutinin(HA)	EPI1836639
A/Duck/Fujian/10.26_FZHX0034-C/2017	H5N6	Hemagglutinin(HA)	EPI1820508
A/Duck/China/FJ17139/2017	H5N6	Hemagglutinin(HA)	EPI1931954
A/Duck/China/FJ17152/2017	H5N6	Hemagglutinin(HA)	EPI1931955
A/Duck/China/FJ1813/2018	H5N6	Hemagglutinin(HA)	EPI1931942

A/Duck/Fujian/11.09_FZHX-O/2017	H5N6	Hemagglutinin(HA)	EPI1820509
A/Duck/China/FJ19179/2017	H5N6	Hemagglutinin(HA)	EPI1931956
A/Muscovy duck/Japan/AQ-HE30-77C2/2018	H5N6	Hemagglutinin(HA)	EPI1771069
A/Duck/China/B2/2018	H5N6	Hemagglutinin(HA)	EPI1890293
A/Duck/China/B3/2019	H5N6	Hemagglutinin(HA)	EPI1890294
A/Duck/China/FJ1922/2019	H5N6	Hemagglutinin(HA)	EPI1931975
A/Duck/China/FJ1923/2019	H5N6	Hemagglutinin(HA)	EPI1931976
A/Duck/China/FJ18262/2018	H5N6	Hemagglutinin(HA)	EPI1931966
A/Goose/Fujian/3.15_FZHX0007-C/2018	H5N6	Hemagglutinin(HA)	EPI1820307
A/Goose/Fujian/3.15_FZHX0007-O/2018	H5N6	Hemagglutinin(HA)	EPI1820304
A/Guangdong/18SF020/2018	H5N6	Hemagglutinin(HA)	EPI1352813
A/Duck/Hunan/1.12_YYGK68H3-OC/2018	H5N6	Hemagglutinin(HA)	EPI1834583
A/Duck/Hunan/1.12_YYGK72H3-OC/2018	H5N6	Hemagglutinin(HA)	EPI1834591
A/Goose/China/Wuhu01/2019	H5N6	Hemagglutinin(HA)	EPI1930771
A/Duck/China/FJ18271/2018	H5N6	Hemagglutinin(HA)	EPI1931967
A/Duck/China/FJ1931/2019	H5N6	Hemagglutinin(HA)	EPI1931977
A/Duck/China/B5/2019	H5N6	Hemagglutinin(HA)	EPI1890296
A/Duck/China/FJ1904/2019	H5N6	Hemagglutinin(HA)	EPI1931971
A/Duck/China/FJ1921/2019	H5N6	Hemagglutinin(HA)	EPI1931974
A/Chicken/Hunan/07.26_YYGK28V3-OC/2018	H5N6	Hemagglutinin(HA)	EPI1821087
A/Duck/Hunan/06.22_YYGK90T3-OC/2018	H5N6	Hemagglutinin(HA)	EPI1821086
A/Duck/China/B6/2019	H5N6	Hemagglutinin(HA)	EPI1890297
A/Duck/China/B8/2019	H5N6	Hemagglutinin(HA)	EPI1890299
A/Duck/China/B7/2019	H5N6	Hemagglutinin(HA)	EPI1890298
A/Duck/China/FJ1915/2019	H5N6	Hemagglutinin(HA)	EPI1931973
A/Duck/China/B1/2018	H5N6	Hemagglutinin(HA)	EPI1890292
A/Duck/China/B4/2019	H5N6	Hemagglutinin(HA)	EPI1890295
A/Duck/China/B9/2019	H5N6	Hemagglutinin(HA)	EPI1890300
A/Duck/Cambodia/e10T241C18/2020	H5N6	Hemagglutinin(HA)	EPI2255526
A/Goose/Fujian/3.15_FZHX0008-C/2018	H5N6	Hemagglutinin(HA)	EPI1836599
A/Duck/Laos/2310/2019	H5N6	Hemagglutinin(HA)	EPI1851224
A/Duck/Hunan/2.06_YYGK78J3-OC/2018	H5N6	Hemagglutinin(HA)	EPI1834927

A/Chicken/Guangdong/G1012/2018	H5N6	Hemagglutinin(HA)	EPI1255952
A/Goose/Fujian/3.15_FZHX0010-O/2018	H5N6	Hemagglutinin(HA)	EPI1820305
A/Duck/China/FJ1807/2018	H5N6	Hemagglutinin(HA)	EPI1931932
A/Duck/China/FJ1808/2018	H5N6	Hemagglutinin(HA)	EPI1931934
A/Goose/Fujian/3.15_FZHX0001-O/2018	H5N6	Hemagglutinin(HA)	EPI1820303
A/Goose/Fujian/3.15_FZHX0008-O/2018	H5N6	Hemagglutinin(HA)	EPI1820535
A/Goose/Fujian/3.15_FZHX0005-C/2018	H5N6	Hemagglutinin(HA)	EPI1820306
A/Goose/Fujian/3.15_FZHX0005-O/2018	H5N6	Hemagglutinin(HA)	EPI1820533
A/Muscovy duck/Japan/AQ-HE30-77C1/2018	H5N6	Hemagglutinin(HA)	EPI1771061
A/Chicken/Fujian/9.24_FZHX0068-C/2018	H5N6	Hemagglutinin(HA)	EPI1820974
A/Chicken/Fujian/9.24_FZHX0067-O/2018	H5N6	Hemagglutinin(HA)	EPI1820973
A/Chicken/Fujian/9.24_FZHX0088-C/2018	H5N6	Hemagglutinin(HA)	EPI1820982
A/Duck/China/FJ18220/2018	H5N6	Hemagglutinin(HA)	EPI1931947
A/Duck/China/FJ1935/2019	H5N6	Hemagglutinin(HA)	EPI1931980
A/Duck/China/FJ18108/2018	H5N6	Hemagglutinin(HA)	EPI1931940
A/Duck/China/FJ18231/2019	H5N6	Hemagglutinin(HA)	EPI1931963
A/Duck/China/FJ1854/2018	H5N6	Hemagglutinin(HA)	EPI1931970
A/Duck/Jiangxi/12.17_NCNP71D4-O/2018	H5N6	Hemagglutinin(HA)	EPI1821081
A/Chicken/Guizhou/10.28_ZYLJJ008-C/2018	H5N6	Hemagglutinin(HA)	EPI1820150
A/Duck/Hainan/12.29_HKPL002-C/2017	H5N6	Hemagglutinin(HA)	EPI1834391
A/Duck/Guizhou/8.26_ZYLJJ017-O/2018	H5N6	Hemagglutinin(HA)	EPI1834359
A/Guangxi/31906/2018	H5N6	Hemagglutinin(HA)	EPI1352805
A/Chicken/Guizhou/8.26_ZYLJJ014-O/2018	H5N6	Hemagglutinin(HA)	EPI1820580
A/Duck/Guizhou/8.26_ZYLJJ016-O/2018	H5N6	Hemagglutinin(HA)	EPI1820127
A/Chicken/Vietnam/HU9-842/2018	H5N6	Hemagglutinin(HA)	EPI1584763
A/Chicken/Vietnam/HU9-847/2018	H5N6	Hemagglutinin(HA)	EPI1584779
A/Chicken/Cambodia/c9T241C17T/2019	H5N6	Hemagglutinin(HA)	EPI2584395
A/Duck/Cambodia/c18MKAP189/2018	H5N6	Hemagglutinin(HA)	EPI2581820
A/Duck/Cambodia/c18MKAP211/2018	H5N6	Hemagglutinin(HA)	EPI2581827
A/Duck/Cambodia/c18MKAP214/2018	H5N6	Hemagglutinin(HA)	EPI2581835
A/Guangxi/32797/2018	H5N6	Hemagglutinin(HA)	EPI1352829
A/Duck/Guizhou/7.27_ZYLJJ017-O/2018	H5N6	Hemagglutinin(HA)	EPI1820121

A/Chicken/Quang Tri/V4S4VTC/2020	H5N6	Hemagglutinin(HA)	EPI1963103
A/Chicken/Thanh Hoa/6081VTC/2020	H5N6	Hemagglutinin(HA)	EPI1963245
A/Duck/Thanh Hoa/1107VTC/2020	H5N6	Hemagglutinin(HA)	EPI1814529
A/Duck/Thanh Hoa/923VTC/2020	H5N6	Hemagglutinin(HA)	EPI1814497
A/Duck/Thanh Hoa/945VTC/2020	H5N6	Hemagglutinin(HA)	EPI1814505
A/Chicken/Thanh Hoa/V3S3VTC/2020	H5N6	Hemagglutinin(HA)	EPI1963151
A/Duck/Thanh Hoa/4643VTC/2020	H5N6	Hemagglutinin(HA)	EPI1963293
A/Muscovy duck/Nghe An/259VTC/2021	H5N6	Hemagglutinin(HA)	EPI1963212
A/Muscovy duck/Nghe An/6873VTC/2020	H5N6	Hemagglutinin(HA)	EPI1963167
A/Chicken/Nghe An/7007VTC/2020	H5N6	Hemagglutinin(HA)	EPI1963182
A/Muscovy duck/Nghe An/7006VTC/2020	H5N6	Hemagglutinin(HA)	EPI1963198
A/Duck/Thanh Hoa/1180VTC/2020	H5N6	Hemagglutinin(HA)	EPI1814553
A/Chicken/Thanh Hoa/1152VTC/2020	H5N6	Hemagglutinin(HA)	EPI1814545
A/Duck/Thanh Hoa/1180VTC/2020	H5N6	Hemagglutinin(HA)	EPI1814553
A/Duck/Thanh Hoa/2202VTC/2020	H5N6	Hemagglutinin(HA)	EPI1814577
A/Duck/Thanh Hoa/1151VTC/2020	H5N6	Hemagglutinin(HA)	EPI1814537
A/Duck/Thanh Hoa/1182VTC/2020	H5N6	Hemagglutinin(HA)	EPI1814561
A/Chicken/Viet Nam/21-369/2021	H5N6	Hemagglutinin(HA)	EPI2147136
A/Chicken/Ha Tinh/514VTC/2021	H5N6	Hemagglutinin(HA)	EPI1963276
A/Chicken/Thanh Hoa/1351VTC/2021	H5N6	Hemagglutinin(HA)	EPI1963228
A/Chicken/Thanh Hoa/13836VTC/2019	H5N6	Hemagglutinin(HA)	EPI1665392
A/Duck/Thanh Hoa/740VTC/2020	H5N6	Hemagglutinin(HA)	EPI1814425
A/Chicken/Nghe An/14475VTC/2020	H5N6	Hemagglutinin(HA)	EPI1814585
A/Duck/Nghe An/14528VTC/2020	H5N6	Hemagglutinin(HA)	EPI1814593
A/Duck/Thanh Hoa/752VTC/2020	H5N6	Hemagglutinin(HA)	EPI1814441
A/Duck/Long An/AI470/2018	H5N6	Hemagglutinin(HA)	EPI1363707
A/Chicken/Nghe An/01VTC/2018	H5N6	Hemagglutinin(HA)	EPI1583304
A/Chicken/Nghe An/27VTC/2018	H5N6	Hemagglutinin(HA)	EPI1583716
A/Duck/Nha Trang/301/2018	H5N6	Hemagglutinin(HA)	EPI1776375
A/Common gull/Saratov/1676/2018	H5N6	Hemagglutinin(HA)	EPI1355418
A/Duck/Nghe An/5382VTC/2019	H5N6	Hemagglutinin(HA)	EPI1665320
A/Muscovy duck/Thanh Hoa/879VTC/2020	H5N6	Hemagglutinin(HA)	EPI1814473

A/Chicken/Thanh Hoa/968VTC/2020	H5N6	Hemagglutinin(HA)	EPI1814513
A/Muscovy duck/Thanh Hoa/892VTC/2020	H5N6	Hemagglutinin(HA)	EPI1814481
A/Chicken/Thanh Hoa/844VTC/2020	H5N6	Hemagglutinin(HA)	EPI1814465
A/Duck/Thanh Hoa/676VTC/2020	H5N6	Hemagglutinin(HA)	EPI1814393
A/Duck/Thanh Hoa/5331VTC/2020	H5N6	Hemagglutinin(HA)	EPI1963260
A/Duck/Thanh Hoa/808VTC/2020	H5N6	Hemagglutinin(HA)	EPI1814449
A/Duck/Thanh Hoa/893VTC/2020	H5N6	Hemagglutinin(HA)	EPI1814489
A/Chicken/Thanh Hoa/980VTC/2020	H5N6	Hemagglutinin(HA)	EPI1814521
A/Chicken/Nghe An/842VTC/2020	H5N6	Hemagglutinin(HA)	EPI1814457
A/Duck/Nghe An/694VTC/2020	H5N6	Hemagglutinin(HA)	EPI1814401
A/Duck/Thanh Hoa/722VTC/2020	H5N6	Hemagglutinin(HA)	EPI1814417
A/Duck/Nghe An/1693VTC/2020	H5N6	Hemagglutinin(HA)	EPI1814569
A/Duck/Nghe An/695VTC/2020	H5N6	Hemagglutinin(HA)	EPI1814409
A/Painted stork/India/10CA03/2016	H5N8	Hemagglutinin(HA)	EPI858844
A/Turkey/Poland/23/2019	H5N8	Hemagglutinin(HA)	EPI1669669
A/Wild bird/Korea/H379/2020	H5N8	Hemagglutinin(HA)	EPI1846666
A/Chicken/Kagawa/11C/2020	H5N8	Hemagglutinin(HA)	EPI1815033
A/Whooper swan/Miyagi/0402B001/2021	H5N8	Hemagglutinin(HA)	EPI1896395
A/Mute swan/Croatia/70/2016	H5N8	Hemagglutinin(HA)	EPI861568
A/Duck/France/161108h/2016	H5N8	Hemagglutinin(HA)	EPI869809
A/Chicken/Penza/300/2018	H5N8	Hemagglutinin(HA)	EPI1270882
A/Wigeon/Italy/17VIR57-3/2017	H5N8	Hemagglutinin(HA)	EPI888088
A/Duck/Egypt/A16793/2019	H5N8	Hemagglutinin(HA)	EPI1901636
A/Chicken/Iraq/1/2020	H5N8	Hemagglutinin(HA)	EPI1811628
A/Mute swan/Norway/FU452_21VIR7634-2/2021	H5N8	Hemagglutinin(HA)	EPI1945554
A/Pigeon/Kazakhstan/15-20-B-Talg-5/2020	H5N8	Hemagglutinin(HA)	EPI1882560
A/Chicken/Kagoshima/B3T/2021	H5N8	Hemagglutinin(HA)	EPI1933687
A/Mallard/Georgia/DT-22362/2020	H5N8	Hemagglutinin(HA)	EPI1924294
A/Mute swan/Finland/1325_21VIR7689-2/2021	H5N8	Hemagglutinin(HA)	EPI1945501
A/Swan/Tumen/1479-2/2020	H5N8	Hemagglutinin(HA)	EPI1814684
A/Chicken/Korea/H2599/2015	H5N8	Hemagglutinin(HA)	EPI1682667
A/Chicken/Netherlands/EMC-3/2014	H5N8	Hemagglutinin(HA)	EPI552776

Supplementary Table 5. Genbank accession numbers of H5-HA_{193D} viruses used in the study

Virus name	subtype	segment	Accession Number
A/Chicken/Hunan/Q23/2009	H5	Hemagglutinin(HA)	HM006797.1
A/Vietnam/1203/2004	H5N1	Hemagglutinin(HA)	AY651334.1
A/Environment/Korea/W149/2006	H5N1	Hemagglutinin(HA)	EU233731.1
A/Environment/Korea/W150/2006	H5N1	Hemagglutinin(HA)	EU233739.1
A/Chicken/Hong_Kong/8825-2/2008	H5N1	Hemagglutinin(HA)	KF169906.1
A/Peregrine falcon/Hong Kong/810/2009	H5N1	Hemagglutinin(HA)	AB521159.1
A/Chicken/Henan/Q4/2007	H5N1	Hemagglutinin(HA)	HM583607.1
A/Pigeon/Hunan/185/2010	H5N1	Hemagglutinin(HA)	MW676300.1
A/Pigeon/Hubei/RP25/2012	H5N1	Hemagglutinin(HA)	KT587286.1
A/Chicken/Gaoyou/1004/2010	H5N1	Hemagglutinin(HA)	KP735811.1
A/Chicken/Jilin/Q23/2009	H5N1	Hemagglutinin(HA)	HM583609.1
A/Chicken/China/k0602/2010	H5N1	Hemagglutinin(HA)	KC631946.1
A/Chicken/North_China/k0604/2010	H5N1	Hemagglutinin(HA)	KC261466.1
A/Chicken/Shandong/k0603/2010	H5N1	Hemagglutinin(HA)	KC631945.1
A/Chicken/Dongtai/601/2011	H5N1	Hemagglutinin(HA)	KP735814.1
A/Chicken/China/JX/2011	H5N1	Hemagglutinin(HA)	KC631944.1
A/Chicken/Jiangsu/LHWK/2010	H5N1	Hemagglutinin(HA)	KP735817.1
A/Chicken/Jiangsu/k0101/2010	H5N1	Hemagglutinin(HA)	KC709803.1
A/Chicken/Xuzhou/1012/2010	H5N1	Hemagglutinin(HA)	KP735808.1
A/Chicken/Jiangsu/WJ/2009	H5N1	Hemagglutinin(HA)	KC631943.1
A/Chicken/Jiangsu/XZ/2010	H5N1	Hemagglutinin(HA)	KC631942.1
A/Duck/Jiangxi/120/2011	H5N1	Hemagglutinin(HA)	MK744007.1
A/Chicken/Zhaozhuang/ZG13/2010	H5N1	Hemagglutinin(HA)	KP735807.1
A/Chicken/Danyang/1105/2011	H5N1	Hemagglutinin(HA)	KP735810.1
A/Chicken/Shanxi/0302/2012	H5N1	Hemagglutinin(HA)	KP735816.1
A/Chicken/Dongtai/1011/2010	H5N1	Hemagglutinin(HA)	KP735809.1
A/Chicken/Eastern China/ZG16/2010	H5N1	Hemagglutinin(HA)	KC261467.1
A/Chicken/Eastern China/ZG56/2011	H5N1	Hemagglutinin(HA)	KC261465.1
A/Chicken/Yangzhou/06/2011	H5N1	Hemagglutinin(HA)	KP735815.1

A/Chicken/Aomori/TU9-41,42/2021	H5N1	Hemagglutinin(HA)	LC699176.1
A/Chicken/Kagoshima/TU2-18,19/2021	H5N1	Hemagglutinin(HA)	LC699648.1
A/Goose/Zhejiang/77166/2014	H5N2	Hemagglutinin(HA)	KU042750.1
A/Duck/Eastern China/S0131/2014	H5N2	Hemagglutinin(HA)	KP732639.1
A/Chicken/Tibet/LZ01/2010	H5N2	Hemagglutinin(HA)	JX565019.1
A/Chicken/Shenzhen/1845/2013	H5N6	Hemagglutinin(HA)	KP284981.1
A/Duck/Sichuan/NCXJ16/2014	H5N6	Hemagglutinin(HA)	KM251466.1
A/Chicken/Sichuan/NCJPL1/2014	H5N6	Hemagglutinin(HA)	KM251463.1
A/Anas crecca/Hubei/Chenhu1623-5/2014	H5N6	Hemagglutinin(HA)	KM251462.1
A/Goose/Zhejiang/925105/2014	H5N6	Hemagglutinin(HA)	KU042759.1
A/Goose/Zhejiang/925108/2014	H5N6	Hemagglutinin(HA)	KU042761.1
A/Duck/Vietnam/NCVD-15A57/2015	H5N6	Hemagglutinin(HA)	KY171721.1
A/Chicken/Japan/AQ-HE144/2015	H5N6	Hemagglutinin(HA)	LC208492.1
A/Duck/Jiangxi/11.17_NCNP30Y2-O/2016	H5N6	Hemagglutinin(HA)	MW108616.1
A/Duck/Jiangxi/11.17_NCNP48Y2-C/2016	H5N6	Hemagglutinin(HA)	MW108624.1
A/Chicken/Zhejiang/528127/2016	H5N6	Hemagglutinin(HA)	MW485699.1
A/Duck/Hunan/03/07_YYGK4L3-OC/2018	H5N6	Hemagglutinin(HA)	MW107912.1
A/Muscovy duck/Vietnam/HU7-20/2017	H5N6	Hemagglutinin(HA)	LC364036.1
A/Muscovy duck/Vietnam/HU7-17/2017	H5N6	Hemagglutinin(HA)	LC364028.1
A/Muscovy duck/Vietnam/HU7-23/2017	H5N6	Hemagglutinin(HA)	LC364044.1
A/Duck/Quang Ninh/371/2018	H5N6	Hemagglutinin(HA)	LC536346.1
A/Duck/Vietnam/LBM1099/2018	H5N6	Hemagglutinin(HA)	LC536306.1
A/Duck/Nha Trang/271/2018	H5N6	Hemagglutinin(HA)	LC536330.1
A/Chicken/Vietnam/Raho4-Cd-20-421/2020	H5N6	Hemagglutinin(HA)	OQ673101.1
A/Duck/Viet Nam/HU13-163/2019	H5N6	Hemagglutinin(HA)	MT107010.1
A/Duck/Viet Nam/HU12-970/2019	H5N6	Hemagglutinin(HA)	MT106954.1
A/Duck/Viet Nam/HU12-982/2019	H5N6	Hemagglutinin(HA)	MT547660.1
A/Duck/Viet Nam/HU12-980/2019	H5N6	Hemagglutinin(HA)	MT107002.1
A/Duck/Viet Nam/HU12-981/2019	H5N6	Hemagglutinin(HA)	MT547638.1
A/Duck/Viet Nam/HU12-977/2019	H5N6	Hemagglutinin(HA)	MT106978.1
A/Duck/Viet Nam/HU12-971/2019	H5N6	Hemagglutinin(HA)	MT106962.1
A/Duck/Viet Nam/HU12-978/2019	H5N6	Hemagglutinin(HA)	MT106986.1

A/Duck/Viet Nam/HU12-972/2019	H5N6	Hemagglutinin(HA)	MT106970.1
A/Duck/Viet Nam/HU12-979/2019	H5N6	Hemagglutinin(HA)	MT106994.1
A/Duck/Eastern China/S0322/2014	H5N6	Hemagglutinin(HA)	KP732644.1
A/Chicken/Vietnam/NCVD-15A59/2015	H5N6	Hemagglutinin(HA)	KY171730.1
A/Quail/Viet Nam/4615/2015	H5N6	Hemagglutinin(HA)	MZ049569.1
A/Common teal/Korea/W558/2017	H5N6	Hemagglutinin(HA)	KY576116.1
A/Environment/Korea/W544/2016	H5N6	Hemagglutinin(HA)	KY273000.1
A/Anas platyrhynchos/South Korea/1702/2017	H5N6	Hemagglutinin(HA)	MN565986.1
A/Wild duck/South Korea/1920/2019	H5N6	Hemagglutinin(HA)	MN577334.1
A/Chicken/Taishun/TS2/2016	H5N6	Hemagglutinin(HA)	KY415633.1
A/Chicken/Taishun/TS90/2016	H5N6	Hemagglutinin(HA)	KY415632.1
A/Duck/Vietnam/HN6611/2020	H5N6	Hemagglutinin(HA)	MW872790.1
A/Muscovy duck/Vietnam/HN6607/2020	H5N6	Hemagglutinin(HA)	MW872935.1
A/Muscovy duck/Vietnam/HN6610/2020	H5N6	Hemagglutinin(HA)	MW872782.1
A/Muscovy duck/Vietnam/HN6608/2020	H5N6	Hemagglutinin(HA)	MW873018.1
A/Muscovy duck/Vietnam/HN6606/2020	H5N6	Hemagglutinin(HA)	MW872901.1
A/Muscovy duck/Vietnam/HN6609/2020	H5N6	Hemagglutinin(HA)	MW872821.1
A/Muscovy duck/Vietnam/HN6120/2020	H5N6	Hemagglutinin(HA)	MW873179.1
A/Muscovy duck/Vietnam/HN6111/2020	H5N6	Hemagglutinin(HA)	MW873338.1
A/Muscovy duck/Vietnam/HN6119/2020	H5N6	Hemagglutinin(HA)	MW873246.1
A/Muscovy duck/Vietnam/HN6115/2020	H5N6	Hemagglutinin(HA)	MW873196.1
A/Muscovy duck/Vietnam/HN6113/2020	H5N6	Hemagglutinin(HA)	MW873143.1
A/Muscovy duck/Vietnam/HN6114/2020	H5N6	Hemagglutinin(HA)	MW873308.1
A/Duck/Vietnam/QN6519/2020	H5N6	Hemagglutinin(HA)	MW873453.1
A/Muscovy duck/Vietnam/LBM1101/2018	H5N6	Hemagglutinin(HA)	LC536314.1
A/Breeder duck/Korea/Gochang1/2014	H5N8	Hemagglutinin(HA)	KJ413834.1
A/Duck/Eastern China/S1109/2014	H5N8	Hemagglutinin(HA)	KP732646.1
A/Green-winged teal/Egypt/871/2016	H5N8	Hemagglutinin(HA)	MF037851.1
A/Green-winged teal/Egypt/877/2016	H5N8	Hemagglutinin(HA)	MF037862.1
A/Guinea fowl/South Africa/17080274/2017	H5N8	Hemagglutinin(HA)	MH165628.1
A/Chicken/South Africa/499723/2018	H5N8	Hemagglutinin(HA)	MN252530.1
A/Cygnus olor/Belgium/1567/2017	H5N8	Hemagglutinin(HA)	MF073917.1

A/Peacock/Belgium/1017/2017	H5N8	Hemagglutinin(HA)	MF073909.1
A/American wigeon/Washington/195205/2014	H5N8	Hemagglutinin(HA)	KU201888.1
A/Chicken/kumamoto/1-7/2014	H5N8	Hemagglutinin(HA)	AB932556.1
A/Mallard/Korea/W452/2014	H5N8	Hemagglutinin(HA)	KJ746111.1
A/Environment/Korea/W483/2015	H5N8	Hemagglutinin(HA)	KX297881.1
A/Environment/Korea/W488/2015	H5N8	Hemagglutinin(HA)	KX297884.1
A/Goose/Taiwan/TNO3/2015	H5N8	Hemagglutinin(HA)	KT388572.1
A/Tundra swan/Tottori/C6nk/2014	H5N8	Hemagglutinin(HA)	LC026477.1
A/American wigeon/California/UCD58P/2015	H5N8	Hemagglutinin(HA)	KY828643.1
